# MMP-13 Regulates Growth of Wound Granulation Tissue and Modulates Gene Expression Signatures Involved in Inflammation, Proteolysis, and Cell Viability

**DOI:** 10.1371/journal.pone.0042596

**Published:** 2012-08-07

**Authors:** Mervi Toriseva, Matti Laato, Olli Carpén, Suvi T. Ruohonen, Eriika Savontaus, Masaki Inada, Stephen M. Krane, Veli-Matti Kähäri

**Affiliations:** 1 Department of Dermatology, University of Turku and Turku University Hospital, Turku, Finland; 2 MediCity Research Laboratory, University of Turku, Turku, Finland; 3 Department of Surgery, Turku University Hospital, Turku, Finland; 4 Department of Pathology, University of Turku and Turku University Hospital, Turku, Finland; 5 Department of Pharmacology, Drug Development and Therapeutics, University of Turku, Turku, Finland; 6 Department of Life Science and Biotechnology, Faculty of Engineering, Tokyo University of Agriculture and Technology, Tokyo, Japan; 7 Center for Immunology and Inflammatory Diseases, Department of Medicine, Harvard Medical School and Massachusetts General Hospital, Boston, Massachusetts, United States of America; Chang Gung University, Taiwan

## Abstract

Proteinases play a pivotal role in wound healing by regulating cell-matrix interactions and availability of bioactive molecules. The role of matrix metalloproteinase-13 (MMP-13) in granulation tissue growth was studied in subcutaneously implanted viscose cellulose sponge in MMP-13 knockout (*Mmp13* ^−/−^) and wild type (WT) mice. The tissue samples were harvested at time points day 7, 14 and 21 and subjected to histological analysis and gene expression profiling. Granulation tissue growth was significantly reduced (42%) at day 21 in *Mmp13* ^−/−^ mice. Granulation tissue in *Mmp13* ^−/−^ mice showed delayed organization of myofibroblasts, increased microvascular density at day 14, and virtual absence of large vessels at day 21. Gene expression profiling identified differentially expressed genes in *Mmp13* ^−/−^ mouse granulation tissue involved in biological functions including *inflammatory response*, *angiogenesis*, *cellular movement*, *cellular growth and proliferation* and *proteolysis*. Among genes linked to angiogenesis, *Adamts4* and *Npy* were significantly upregulated in early granulation tissue in *Mmp13^−/−^* mice, and a set of genes involved in *leukocyte motility* including *Il6* were systematically downregulated at day 14. The expression of *Pdgfd* was downregulated in *Mmp13* ^−/−^ granulation tissue in all time points. The expression of matrix metalloproteinases *Mmp2, Mmp3, Mmp9* was also significantly downregulated in granulation tissue of *Mmp13* ^−/−^ mice compared to WT mice. *Mmp13* ^−/−^ mouse skin fibroblasts displayed altered cell morphology and impaired ability to contract collagen gel and decreased production of MMP-2. These results provide evidence for an important role for MMP-13 in wound healing by coordinating cellular activities important in the growth and maturation of granulation tissue, including myofibroblast function, inflammation, angiogenesis, and proteolysis.

## Introduction

Wound repair is a fundamental process for survival of multicellular organisms. Mammalian wound healing consists of functionally distinct and temporally overlapping processes, *i.e.* hemostasis and inflammation, re-epithelialization and granulation tissue formation, and tissue remodeling [1]. These processes involve functions of multiple cell types in distinct tissue compartments, and they are strictly orchestrated by various growth factors, cytokines and extracellular matrix (ECM) components [1], [2].

Proteolytic activity plays a pivotal role in cutaneous wound repair [3], [4]. The main classes of proteinases in wound are serine proteinases of plasminogen activator-plasmin system and matrix metalloproteinases (MMPs). MMPs are a family of Zn-dependent endopeptidases, which as a group can cleave a multitude of ECM proteins and non-matrix proteins, including other proteinases, proteinase inhibitors, growth factors, cytokines, and cell surface receptors [3], [4]. In addition, members of ADAM (a disintegrin and metalloproteinase domain) and ADAMTS (a disintegrin and metalloproteinase domain with thrombospondin motifs) families, including ADAM-9 and ADAMTS-1, have been implicated in wound healing [5], [6].

The expression of MMPs in intact skin is low, but cellular stimuli generated by cutaneous injury result in induction of the expression of several MMPs, and MMP activity in general is essential for normal wound healing [3], [4], [7], [8]. Initially, certain MMPs (MMP-1, MMP-2) are released to injured tissue by aggregating platelets [3], [4]. MMPs expressed by cells in human acute cutaneous wounds include collagenase-1 (MMP-1), collagenase-2 (MMP-8) [9], [10], gelatinase-A (MMP-2), gelatinase-B (MMP-9) [11], stromelysin-1 (MMP-3), stromelysin-2 (MMP-10) [12], MT1-MMP [11], MMP-19 [13], MMP-26 [14], and MMP-28 [15]. In adult human skin wounds, MMP-1 is expressed by keratinocytes at the epithelial tip and by fibroblasts in the granulation tissue [9]. MMP-1 is required for keratinocyte migration on collagen, which is an important feature in the initiation of re-epithelialization [16]. In addition, MMP-1 has been suggested to mediate collagen remodeling during wound healing and wound bed maturation [12], [17]. In murine skin, MMP-1 is substituted by mouse interstitial collagenase MMP-13, a close structural homologue of human MMP-13 (collagenase-3) [18]. The tissue specific expression pattern of mouse MMP-13 indicates functional homology between mouse MMP-13 and both human MMP-1 and MMP-13 [9], [19]–[22].

The expression of MMP-13 is not detected in normally healing human adult skin wounds, but abundant expression of MMP-13 by fibroblasts in chronic cutaneous ulcers has been documented [23]. In contrast, the expression of human MMP-13 by fibroblasts has been noted in normal human gingival and fetal skin wounds characterized by scarless wound healing [24], [25]. MMP-13 has been shown to enhance the remodeling of 3-dimensional (3D) collagen matrix, cell morphology and cell viability of dermal fibroblasts *in vitro* [26]. However, the mechanistic role of MMP-13 in wound granulation tissue growth and remodeling *in vivo* is not clear [27], [28].

In this study, we have investigated the role of MMP-13 specifically in the formation of wound granulation tissue. We have utilized a well defined model of experimental granulation tissue induced by viscose cellulose sponge (VCS) in MMP-13 knockout (*Mmp13^−/−^*) mice [29]. The results showed a marked delay in granulation tissue growth in *Mmp13* ^−/−^ mice accompanied with a delay in organization of myofibroblasts and formation of large blood vessels. Using global gene expression profiling we identified sets of differentially expressed genes in *Mmp13* ^−/−^ and wild type (WT) mouse granulation tissue involved in cellular processes such as *cellular movement*, *inflammatory response*, *cellular growth and proliferation*, as well as *cell death* and *proteolysis*. Among genes involved in angiogenesis, *Adamts4* and *Npy* were specifically upregulated in early granulation tissue of *Mmp13* ^−/−^ mouse characterized by increased microvessel density, and *Pdgfd* was generally downregulated. Also inflammatory cytokine gene *Il6* was downregulated in early *Mmp13* ^−/−^ granulation tissue. These results provide evidence for a pivotal role for MMP-13 in regulating cellular functions important in the growth of granulation tissue, including myofibroblast function, angiogenesis, inflammation, and proteolysis.

## Materials and Methods

### Ethics statement

All mouse experiments were performed according to institutional guidelines of the University of Turku and with the permission of the animal test review board of the Regional Government of Southern Finland.

### Experimental mouse granulation tissue model

The establishment of experimental granulation tissue was performed as previously described [30], [31]. Wild type (WT) and MMP-13 knockout (*Mmp13* ^−/−^) mice (males, age 5.5–8 wk) [29], were anesthetized with Hypnorm-Dormicum solution. A single vertical incision was made to dorsal skin under aseptic conditions, a sterile rectangular viscose cellulose sponge (VCS; Cellomeda Oy, Turku, Finland) of size 5×5×10 mm^3^ was implanted horizontally under the skin at cranial location, and the wound was closed with continuous monofilament 4-0 suture (Prolene; Ethicon, Norderstedt, Germany). Both *Mmp13* ^−/−^ and WT mice recovered well from surgical VCS implantation and during the experiment, no wound infections or other complications were observed. The mice (n = 5–6/group) were sacrificed 7, 14, and 21 days after implantation, the sponges were removed and cut into four pieces. Two inner parts of VCS were either frozen with Tissue-Tek O.C.T. Compound (Sakura, Finetek) in liquid nitrogen or fixed in 10% neutral buffered formalin for 24 h and embedded in paraffin for histological assessment. The remaining parts of the rectangular VCS were frozen in liquid nitrogen for RNA extraction (Qiagen RNeasy kit, Qiagen GmbH, Hilden, Germany).

### Histological and morphometrical analysis of mouse granulation tissue

Formalin-fixed, paraffin-embedded tissue sections were processed for hematoxylin and eosin (HE) staining and subjected for microscopic evaluation. The mosaic images of representative samples were obtained using Zeiss Axiovert 200 M microscope with 10× objective and Axiovision 4.3 software (Carl Zeiss MicroImaging GmbH, Germany). To quantify the tissue growth inside VCS HE-stained tissue sections of all samples were scanned. The portion of cellular area of the total implant area was determined utilizing image analysis with cell^D^ 2.6 software (Olympus Soft Imaging Solutions GmbH) and performed as blind analysis. The capsule composed of loose connective tissue present occasionally around the implant was not included in the measured area. To assess deposition of collagen and other fibrous ECM by granulation tissue cells, tissue sections were stained with van Gieson and Gomori's staining methods. The statistical analyses were performed using Independent samples T-test with SPSS 16.0 software.

### Immunohistochemistry

Formalin-fixed paraffin-embedded sections were rehydrated and processed for immunohistochemical (IHC) staining with biotin-streptavidin-peroxidase complex (ABC) –based visualization system (Vectastain ABC Kit; Vector Laboratories, Inc. Burlingame, CA, USA) and using diaminobenzidine (DAB) as substrate, as previously described [32]. The primary antibodies against mouse CD34 (0.4 µg/ml; MEC 14.7, Santa Cruz) and α-smooth muscle actin (α-SMA) (1∶2000; 1A4, A2547, Sigma) were used. For negative control staining, the primary antibodies were omitted and replaced by blocking reagent.

The orientation of α-SMA-positive myofibroblasts was assessed by scoring as follows: weak (+), moderate (++) and strong (+++) (Table 1). Statistical significance was determined using Pearson's χ^2^-test. Blood vessel formation was assessed by determining the density of CD34 positive vessel structures in a defined area of each sample. The diameter of vessels was morphometrically analyzed with image analysis using cell^D^ 2.6 software, and the vessels were divided into subgroups based on their diameter. Statistical significance was determined using Mann-Whitney U test.

**Table 1:** Evaluation of myofibroblast orientation in WT and *Mmp13^−/−^* mouse granulation tissue.

Time point	Genotype	+	++	+++	*p-value*
**7 d**	WT	0	2	3	<0.05
	*Mmp13^−/−^*	3	3	0	
**14 d**	WT	0	2	4	n.s.
	*Mmp13^−/−^*	0	2	3	
**21 d**	WT	3	2	0	<0.05
	*Mmp13^−/−^*	0	2	3	

Sections of experimental granulation tissue of wild-type (WT) and MMP-13 knockout (*Mmp13^−/−^*) mice were stained for myofibroblasts with α-SMA antibody and analyzed for myofibroblast orientation. Scoring: +, weak; ++, moderate; +++, strong. Scoring is based on the parallel orientation of myofibroblasts to the implant surface where *weak* implies negligible orientation, *moderate* implies lining of occasional myofibroblasts in certain areas, and *strong* indicates intensive parallel lining of myofibroblast masses. Statistical significance (*p*) was determined by Pearson's χ^2^-test. n.s., not significant.

### Gene expression profiling of mouse granulation tissue

Genome wide gene expression profiling in mouse experimental granulation tissue (day 7, n = 3; day 14, n = 4; day 21, n = 4 for both genotypes) with Affymetrix GeneChip® 3′ IVT Expression Analysis was performed at The Finnish DNA Microarray and Sequencing Centre, Turku, Finland. Aliquots of total RNA were processed according to Affymetrix's instructions and hybridized to Mouse Genome 430 2.0 oligonucleotide array (Affymetrix, Santa Clara, CA). The data quality was checked with AGCC and Expression Console™ 1.1. The data were normalized between the chips using rma and subjected for statistical testing with Chipster v1.4.7 software (CSC - IT Center for Science Ltd., Espoo, Finland). Statistical significances for differentially expressed genes were determined using linear modeling. The gene expression pattern was evaluated as comparison of the genotypes in a given time point. Moreover, differentially expressed genes were determined between two consecutive time points in WT samples. The fold change (FC) value for differentially expressed genes is essentially the log2 of the ratio between the mean expression values of the two sample groups. Further data mining was performed using Ingenuity Pathway Analysis (IPA®) software (http://www.ingenuity.com/). The data sets from genotype comparisons in each time point were analyzed using threshold *P*<0.05 and FC>0.5, which was chosen based on volcano plot data visualization. Illustration of the data was performed with IPA® and RGui v2.11.1 software. All microarray data are MIAME compliant and have been deposited in the public database GEO (Gene Expression Omnibus, NCBI; accession number GSE38822).

### Real-time quantitative RT-PCR

For quantitative analysis of the selected mRNAs aliquots of total RNA were DNase-treated, reverse-transcribed to cDNA and analyzed using real-time quantitative RT-PCR (qRT-PCR) with TaqMan® technology and Applied Biosystem's 7900HT equipment [33]. For mouse neuropeptide Y (*Npy*) mRNA, pre-designed TaqMan® Gene Expression assay was used (ID Mm03048253_m1, Applied Biosystems). For other transcripts, oligonucleotide primers and dual-labeled probes were designed using RealTimeDesign™ software (Biosearch Technologies, Inc) (Table S1). The levels of β-actin mRNA were used to normalize the results between the samples. The samples were analyzed in three technical replicates. To determine statistical significance of the results, the data were analyzed with independent samples T-test.

### Culture of mouse skin fibroblasts

WT and *Mmp13^−/−^* mouse [29] skin fibroblasts (MSF) from three individual mice for each genotype were established by explantation from the skin of 3 weeks old male and female mice. Dorsal skin pieces (1×1 mm) were allowed to attach on cell culture dish for 10 min and covered with DMEM containing heat-inactivated fetal calf serum (FCS, 20%), L-glutamine (2 mM), penicillin G (150 IU/ml), streptomycin (150 µg/ml) and Amphotericin B (1 µg/ml). After 10 days of cultivation, the skin pieces were removed and the fibroblasts cultured until subconfluency. WT and *Mmp13^−/−^* fibroblasts were seeded and cultured in 3D collagen gel, as previously described [26]. The cells were suspended in bovine collagen suspension consisting of 7/10 PureCol® (97% type I atelocollagen, 3 mg/ml, Advanced BioMatrix), 1/10 NaOH in 0.2 M Hepes buffer pH 8 and 1/5 5×DMEM in density 5×10^5^ cells/ml for contraction assay and 2×10^5^ cells/ml for visualizing cell morphology, and 300 µl aliquots were applied into wells of 24-well plate. After solidification (1–2 h, 37°C), the gels were detached from the well edges and DMEM containing 0.5% or 10% FCS was added. In certain cultures, medium was supplemented with transforming growth factor-β (TGF-β) (5 ng/ml). Collagen gel contraction was assessed after 24 h and 48 h by quantifying the gel areas using cell^D^ 2.6 image analysis software. Alternatively, the gel was left attached in the well, released after 72 h, and the contraction was assessed 24 h later. To visualize cell morphology, the gels were fixed with 4% paraformaldehyde (37°C) after 24 h cultivation, stained with fluorescently labeled phalloidin and Hoechst 33342, and examined in a microscope.

### Gelatinase zymography

Aliquots of unheated conditioned media were fractionated in 10% SDS-PAGE containing 1 mg/ml gelatin (G-6269, Sigma) which was fluorescently labeled with MDPF (645989, Fluka). The gels were washed and subsequently incubated for 48 h in a gelatinase activating buffer as described in [34], and photographed under UV-light.

## Results

### Delayed granulation tissue growth in *Mmp13^−/−^* mice

**Figure 1:**
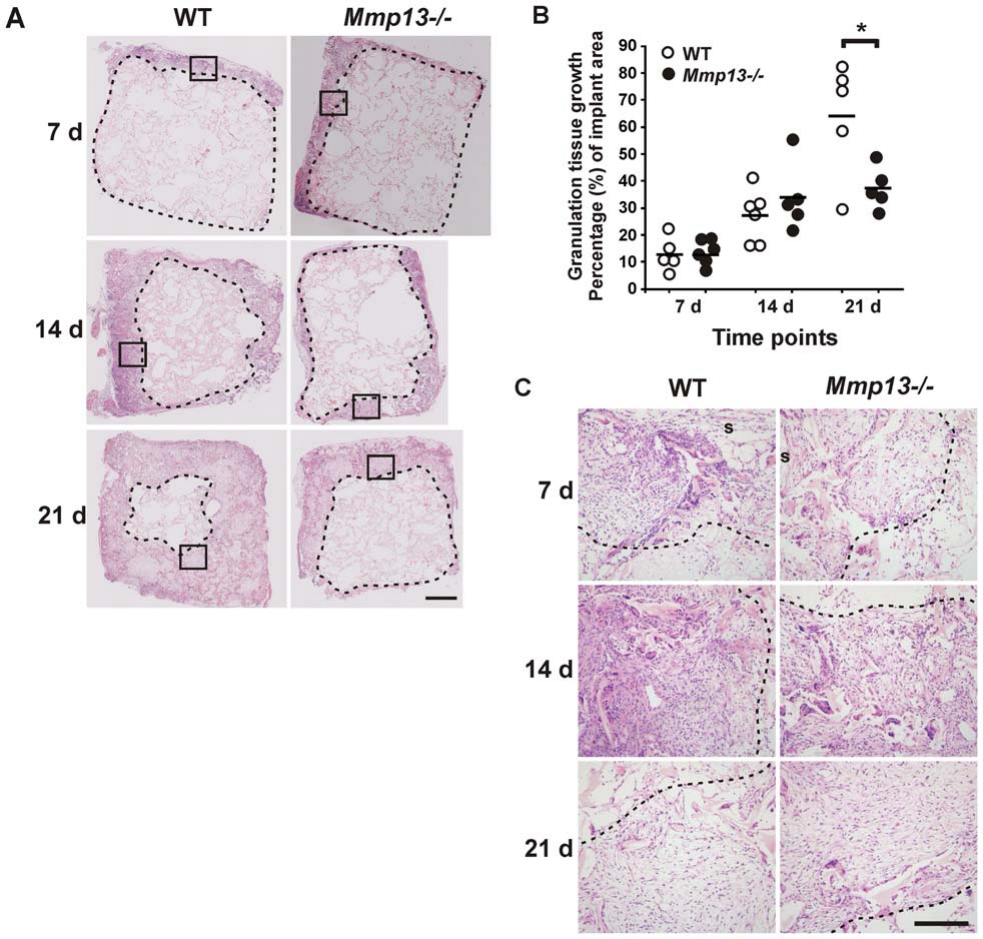
Delayed growth of experimental granulation tissue in *Mmp13^−/−^* mice. Subcutaneous viscose cellulose sponges (VCS) implanted in wild type (WT) and MMP-13 knockout (*Mmp13^−/−^*) mice were harvested at different time points, as indicated. (**A**) Hematoxylin-eosin staining of representative sections demonstrating reduced growth of granulation tissue in *Mmp13^−/−^* mice at 21 d. The border of cellular granulation tissue is marked with dashed line. The area enclosed by a square is shown in (C) with higher magnification. (Scale bar = 1 mm). (**B**) The growth of granulation tissue inside VCS was quantified blinded by determining the portion of cellular tissue relative to the implant area in a tissue section. The border of granulation tissue was determined as exemplified with dashed lines in (A). (*P<0.05, Independent samples T-test, n = 5–6). (**C**) Higher resolution images from the tissue sections presented in (A) showing the border region at the endpoint of the granulation tissue (the area enclosed by a square in A). (s, implant surface; scale bar = 200 µm).

To elucidate the role of MMP-13 specifically in the formation of wound granulation tissue involved in the wound healing process, subcutaneously implanted viscose cellulose sponge (VCS) was used to induce granulation tissue growth. This model has been well characterized and shown to be comparable to the formation of granulation tissue during cutaneous wound healing [30], [31]. Histological analysis revealed similar initial growth of granulation tissue into VCS at 7 d and 14 d in WT and *Mmp13* ^−/−^ mice, characterized by influx of inflammatory cells and fibroblast-like cells, and ingrowth of vessel structures (Figure 1A and C). Van Gieson staining for ECM fibers indicated similar ECM deposition in *Mmp13* ^−/−^ and WT mice adjacent to the implant surface (data not shown). However, at 21 d, the *Mmp13^−/−^* granulation tissue was clearly different from WT tissue as demonstrated by a significant reduction (42%, *P*<0.05) in the growth of granulation tissue in *Mmp13* ^−/−^ mice compared to WT mice at 21 d (Figure 1A and B). The result suggests an important role for MMP-13 in regulating the cellular events related to granulation tissue formation, especially in the later phase characterized by deeper tissue growth.

### Delayed maturation of myofibroblasts in *Mmp13* ^−/−^ mouse granulation tissue

**Figure 2:**
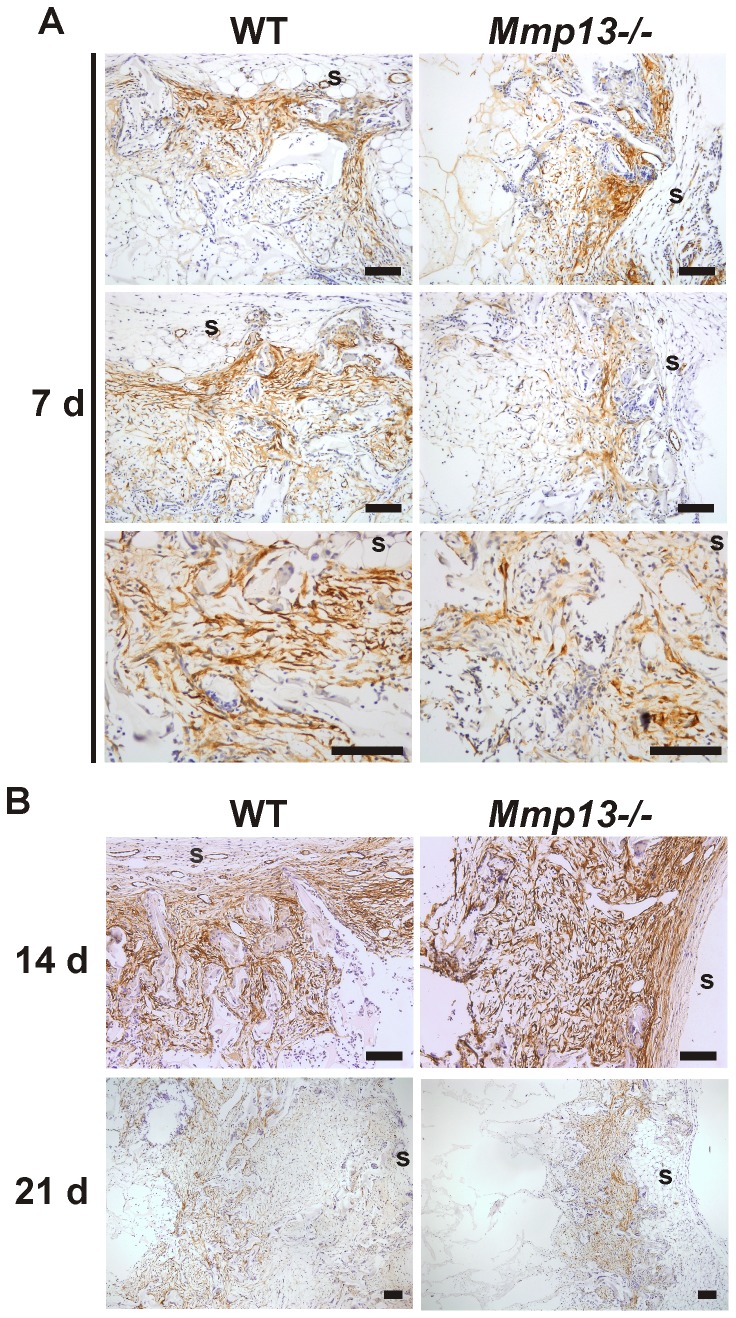
Delayed maturation of myofibroblasts in granulation tissue of *Mmp13^−/−^* mice. Sections of experimental granulation tissue of wild type (WT) and MMP-13 knockout (*Mmp13^−/−^*) mice were stained with α-smooth muscle actin (α-SMA) antibody. (**A**) The panel shows three representative image pairs from comparable locations of WT and *Mmp13^−/−^* granulation tissue at 7 d. α-SMA-positive myofibroblasts were detected close to implant surface (s). The staining pattern was denser and followed parallel orientation more strictly in WT mice compared to *Mmp13^−/−^* granulation tissue. (**B**) (Upper panels) at 14 d, α-SMA-staining pattern was strong and comparable in WT and *Mmp13^−/−^*. (Lower panels) representative image pair of WT and *Mmp13^−/−^* granulation tissues at 21 d immunostained for α-SMA. The expression of α-SMA was evident in the inner parts of implants in WT mouse granulation tissue, whereas in the *Mmp13^−/−^* granulation tissue α-SMA-positive cells were mainly abundant close to implant surface. (s, implant surface; scale bar = 100 µm).

The appearance of myofibroblasts in wound granulation tissue is important for wound contraction during epithelial repair [35]. To assess the presence of myofibroblasts in mouse experimental granulation tissue, sections harvested at 7, 14 and 21 d, were stained for α-SMA by IHC. At 7 d, α-SMA positive cells were detected in the areas adjacent to implant surface in WT mouse tissue and the staining pattern was typically dense and oriented parallel to the surface in accordance with the contractile function of myofibroblasts. In *Mmp13* ^−/−^ mice the orientation of α-SMA-positive myofibroblasts was more random than in WT mice and did not display unified assembly of myofibroblast masses at 7 d. A semi-quantitative evaluation of the staining revealed a significant difference in the collective parallel orientation at 7 d, suggesting altered function and delayed maturation of myofibroblasts (Figure 2A and Table 1). Analysis of the granulation tissue harvested at 14 d showed prominent staining pattern of α-SMA positive cells extending throughout the cellular area and showing strong parallel orientation of myofibroblasts especially in the areas close to VCS surface (Figure 2B). No obvious difference was noted between *Mmp13^−/−^* and WT tissues, suggesting that although lack of MMP-13 results in delayed maturation of myofibroblasts in the granulation tissue, this effect is subsequently compensated by other factors. Interestingly, at 21 d the α-SMA staining pattern was characterized by markedly diminished number of α-SMA-positive cells in the areas close to VCS surface apparently representing the most mature granulation tissue, and the α-SMA staining was more emphasized in the inner parts of the implant. The shift in the expression pattern of α-SMA was clearly more evident in WT than in *Mmp13* ^−/−^ tissue and appeared to be in accordance with the intensive tissue ingrowth (Figure 2B).

### Altered vascularization in the granulation tissue of *Mmp13^−/−^* mice

**Figure 3:**
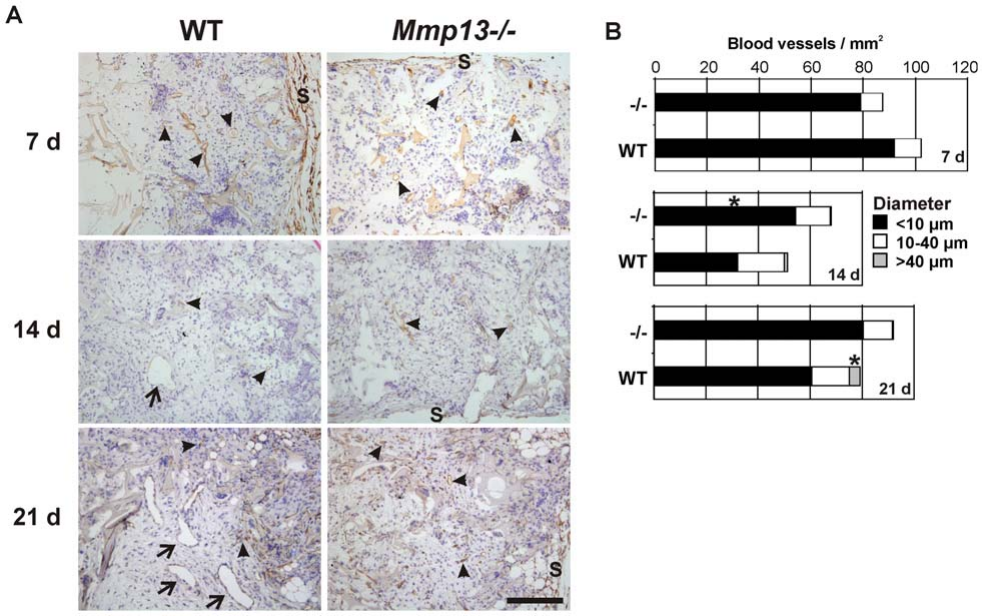
Altered vascular pattern in granulation tissue of *Mmp13^−/−^* mice. (**A**) Sections of experimental granulation tissue of wild-type (WT) and MMP-13 knockout (*Mmp13^−/−^*) mice harvested at indicated time points were immunostained for blood vessels using CD34 as a marker. The *arrowheads* indicate microvessels and medium sized vessels (diameter<40 µm) and *arrows* indicate large vessel structures (diameter>40 µm). (s, implant surface; scale bar = 200 µm. (**B**) The number and the diameter of CD34-positive blood vessels were determined in defined areas of cellular granulation tissues with digital image analysis. *Statistically significant difference in the density of microvessels (<10 µm) at 14 d and of the large vessels (>40 µm) at 21 d (*P*<0.05, MannWhitney U test, n = 5–6).

To examine the role of MMP-13 in vascularization of the experimental granulation tissue, the tissue sections were stained for CD34 by IHC. The CD34-positive vessels were morphometrically analyzed and subdivided into three groups based on the diameter. The vessel structures with the diameter less than 10 µm were considered as microvessels, the vessels with the diameter 10–40 µm as medium sized blood vessels, and the vessels over 40 µm in diameter as large vessels. In general, CD34 positive blood vessels were abundantly present in WT and *Mmp13* ^−/−^ mouse granulation tissue already at 7 d time point in the areas with prominent tissue growth (Figure 3A). In both groups, the vessel density decreased during the second week of granulation tissue growth and increased again during the third week. CD34 positive microvessels (<10 µm) were abundantly present in both WT and *Mmp13* ^−/−^ mouse granulation tissue in all time points examined (Figure 3B). Interestingly, microvessel density was higher in *Mmp13* ^−/−^ mouse granulation tissue at 14 d (*P*<0.05), reflecting enhanced neovascularization or reduced resolution. The density of the medium sized vessels (10–40 µm) was similar in WT and *Mmp13^−/−^* tissue in all time points examined (Figure 3A and B). A striking difference in vascular pattern was also noted at 21 d, when the *Mmp13* ^−/−^ granulation tissue was characterized by almost total absence of large vessels (>40 µm), likely to represent venules or arterioles, which in contrast, were commonly present in WT mouse granulation tissue (Figure 3A and B) (*P*<0.05). Determination of the total number of large vessels at 21 d time point revealed that a section of every granulation tissue sample contained in average 14 (range 5–24) and 2 (range 0–4) large vessels in WT and *Mmp13* ^−/−^ mouse granulation tissue, respectively.

### Comparison of gene expression profiles between *Mmp13^−/−^* and WT mouse granulation tissue

**Figure 4:**
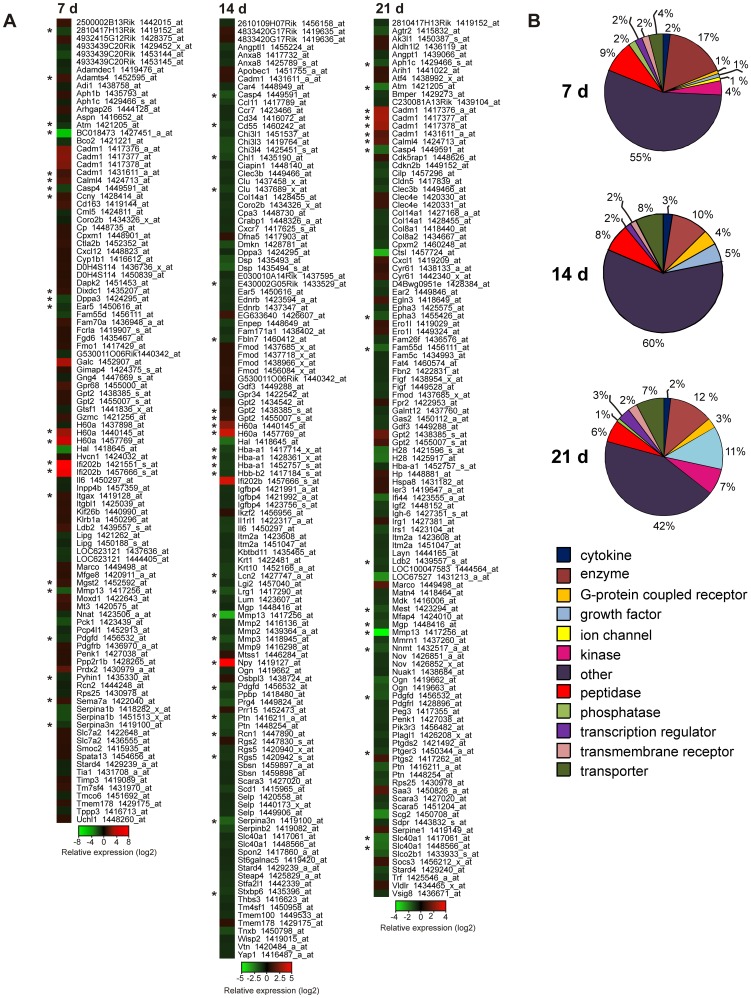
Comparison of gene expression profiles in granulation tissue of *Mmp13^−/−^* and WT mice. (**A**) Microarray data of MMP-13 knockout (*Mmp13^−/−^*) and wild type (WT) mouse granulation tissue at 7, 14 and 21 d were analyzed for differential gene expression by comparing *Mmp13^−/−^* granulation tissue samples to WT. The genes, which showed significant difference (*P*<0.05) and FC>0.75 in the expression are illustrated as heatmap. *Genes with FC>1 and *P*<0.001. (**B**) Differentially expressed genes at indicated time points were categorized based on molecular function according to Ingenuity Pathway Analysis® (IPA) software.

To gain insight into the molecular mechanisms of MMP-13 -elicited regulation of granulation tissue growth and vascularization, RNAs from WT and *Mmp13^−/−^* mouse granulation tissue samples were subjected to oligonucleotide microarray (Affymetrix) based global gene expression profiling. The data were analyzed by comparison of the gene expression profiles of *Mmp13^−/−^* and WT tissue in all time points. Here, 1303, 3560 and 1984 significantly differentially expressed genes (*P*<0.05) were identified at 7 d, 14 d and 21 d, respectively. Of these 87, 96 and 95 genes, respectively, displayed FC>0.75 and are listed in heatmap in Figure 4A.

Classification of differentially expressed genes between the genotypes at specific time points based on the molecular function revealed the peptidases and other enzymes and the group of unclassified genes (“other”) as the largest groups at 7 d (Figure 4B). The majority of the differentially expressed transcripts at 7 d were upregulated in *Mmp13* ^−/−^ mouse granulation tissue, including remarkable upregulation of interferon activated gene 202B (*Ifi202b*, *p202*; FC∼8) and cell adhesion molecule 1 (*Cadm1*; FC∼2.5), which both are associated with negative regulation of cell growth [37], [38]. Interestingly, the expression of minor histocompatibility antigen 60a (*H60a*) was markedly upregulated in *Mmp13^−/−^* tissue at 7 d and 14 d (FC∼4 and FC∼3, respectively). In addition to upregulation of *Adamts4* and down-regulation of serine/cysteine peptidase inhibitors *Serpina1b* and *Serpina3n* in *Mmp13* ^−/−^ mice, no other signs of positive regulation of endopeptidase activity, which could potentially compensate MMP-13 deficiency, was recognized. It is of note that *Il6* which codes for proinflammatory cytokine IL-6, an important regulator of the acute-phase response to injury and infection [39], was downregulated in *Mmp13* ^−/−^ tissues at both 7 d and 14 d (FC∼1 and FC∼1.2, respectively).

Interestingly, potent and significant upregulation of the expression of neuropeptide Y (*Npy*; FC∼5), a positive regulator of angiogenesis [40]–[42] was observed in *Mmp13* ^−/−^ tissue as compared to WT mice at 14 d. In addition, expression of *H60a*, *Cadm1* and *Ifi202b* genes remained elevated. In contrast, a variety of genes associated with collagen metabolism and fibrillogenesis (*Mmp2, Mmp3, Mmp9, Tnxb*/tenascin XB, *Col14a1*/collagen type XIV α1) and cell adhesion and motility, and angiogenesis (*e.g. Thbs3*/thrombospondin 3, *Dsp*/desmoplakin, *Chl1*/cell adhesion molecule with homology to L1CAM, *Selp*/selectin P, *Vtn*/vitronectin, *Pdgfd*/platelet-derived growth factor D, *Tnxb*, and *Mmp9*) were down-regulated in *Mmp13* ^−/−^ granulation tissue (Figure 4A). Interestingly, the expression of *Hba-a1*/hemoglobin α-chain previously detected in granulation tissue macrophages [43] was downregulated in *Mmp13* ^−/−^ tissue at 14 d.

At 21 d, over 10% of the differentially expressed genes were growth factors, all downregulated in *Mmp13* ^−/−^ tissue (Figure 4B). In addition, several genes associated with angiogenesis (*e.g. Col8a1, Col8a2*/collagen VIII α1 and α2, *Angpt1*/angiopoietin 1 and *Figf*/c-fos induced growth factor/vascular endothelial growth factor D) were downregulated in *Mmp13^−/−^* granulation tissue as compared to WT mice (Figure 4A). Moreover, genes such as *Igf2*/insulin-like growth factor 2, *Ptn*/pleiotrophin, *Pdgfd*, and *Figf*, which function in the positive regulation of cell division were downregulated with FC>0.75 and *P*<0.05. *Casp4*/caspase 4, one of the “inflammatory caspases” and *Pdgfd*, a potent inducer of cell proliferation and angiogenesis [44], [45], were significantly downregulated (FC>1, *P*≤0.001) in all time points examined in *Mmp13* ^−/−^ mice. In contrast, *Cadm1* which is shown to regulate epidermal wound healing [46] was systematically upregulated in all time points (Figure 4A).

### Pathway analysis of differentially expressed genes in *Mmp13* ^−/−^ and WT mouse granulation tissue

**Figure 5:**
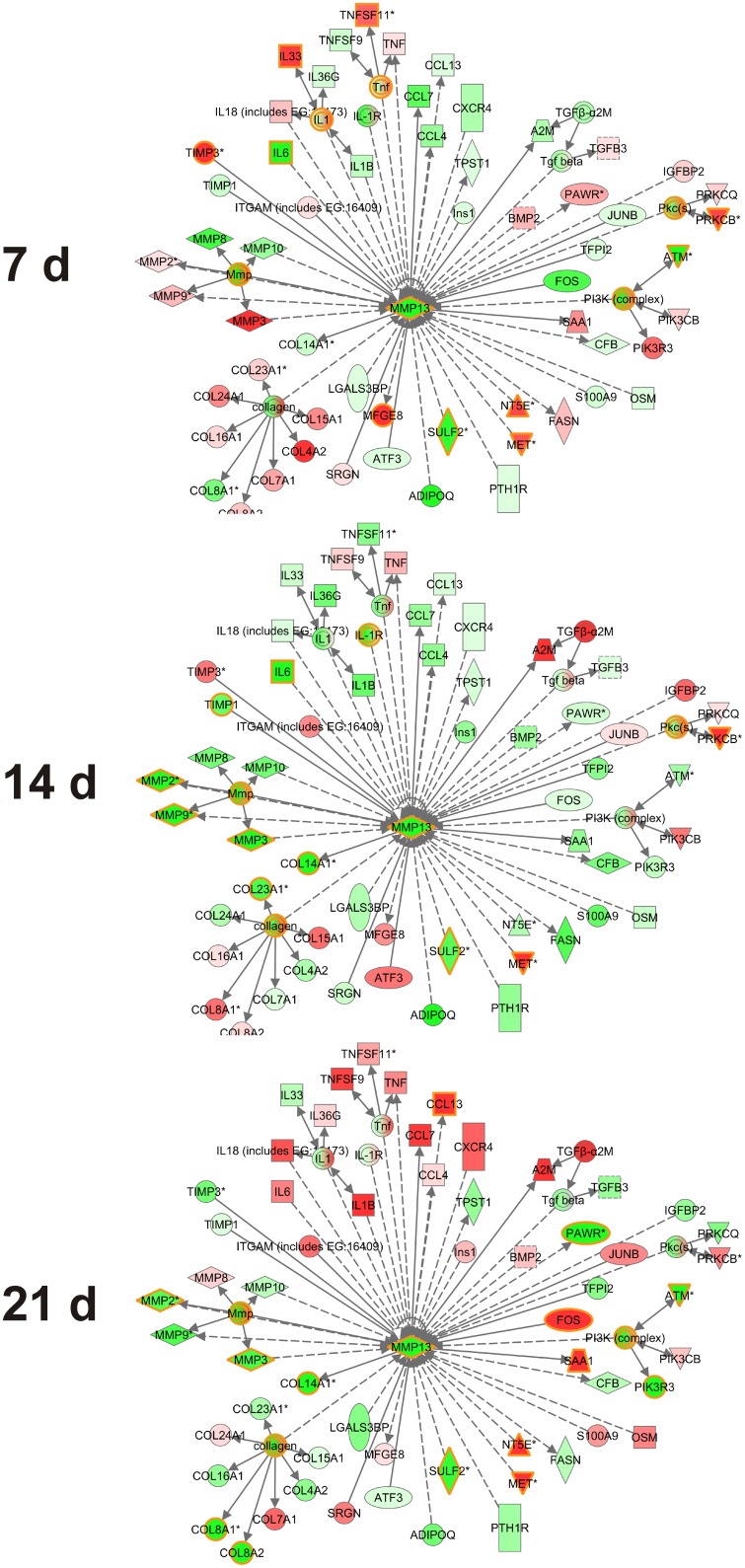
Molecular interactions of MMP-13 with differently regulated genes in *Mmp13^−/−^* mouse granulation tissue compared to WT. IPA software was employed to construct a molecular interaction network of MMP-13 with the genes that were differently expressed in MMP-13 knockout (*Mmp13^−/−^*) granulation tissue compared to wild type (WT) in indicated time points. Interactions are based on the literature in Ingenuity Knowledge Base. The molecules with fold change (FC) >0.3 in one of the time points were included in the figure and the molecules with FC>0.5 and with *P*-value<0.05 in specific time point are highlighted with yellow color. Red color indicates upregulation and green color indicates downregulation in *Mmp13^−/−^* mouse granulation tissue compared to WT. The intensity of the color implies the magnitude of FC. The arrows and lines indicate direct (solid line) and indirect (dashed line) functional and physical interactions. The arrows show the direction of the regulation.

To elucidate the biological processes involved in the delayed granulation tissue growth in *Mmp13^−/−^* mice, the different gene expression profiles at specific time points were subjected to Ingenuity Pathway Analysis (IPA). This analysis of molecular relations was performed based on the genes that differed between the data sets with FC>0.5 and *P*<0.05. Several of the differentially expressed genes included in the gene lists in Figure 4 were also found in the MMP-13 interaction network including *Col8A1, Col8A2* and *Col14A1* and several MMPs. Moreover, the expression of a variety of chemokines and cytokines directly related to MMP-13, including *Il6*, *Il1*, *Ccl7*, *Ccl4* and *Ccl13*, was downregulated at 7 d and 14 d in *Mmp13* ^−/−^ tissue but upregulated at 21 d (Figure 5).

Next, functional analysis was performed to associate biological functions with the differentially expressed gene sets at 7 d, 14 d and 21 d of *Mmp13* ^−/−^ granulation tissue compared to WT (Tables 2, 3, 4). Briefly, at 7 d the differentially expressed molecules (FC>0.5, *P*-value<0.05) were associated most significantly with categories *cellular growth and proliferation*, *cellular movement*, *inflammatory response*, and *cell death* (Table 2). However, based on regulation z-score, significant up- or downregulation of biological functions between genotypes at 7 d was noted only with respect to *neuronal cell death* and *neoplasia* (Table 2). The biofunction *vasculogenesis* was predicted to be upregulated in *Mmp13* ^−/−^ granulation tissue (regulation z-score 1.9). This is in accordance with the increased density of microvessels noted in *Mmp13* ^−/−^ granulation tissue at 14 d by IHC (Figure 3).

**Table 2:** Summary of statistically significant biofunctions associated with the molecules that are differently regulated in *Mmp13^−/−^* granulation tissues at day 7 compared to the corresponding WT samples (IPA Functional Analysis).^1^

*Category* 2	*Function Annotation*	*p-value* 3	*Number of Molecules*	*Regulation z-score* 4
**Cellular Growth and Proliferation**	proliferation of cells	2.19E-13	71	1.266
	growth of fibroblast cell lines	8.86E-04	9	−1.463
	proliferation of smooth muscle cells	1.50E-03	8	1.160
**Cellular Movement**	migration of cells	8.01E-12	51	−0.369
	leukocyte migration	6.48E-08	28	0.204
	migration of endothelial cells	4.86E-07	13	1.226
	chemotaxis	1.11E-06	19	0.929
**Inflammatory Response**	immune response	3.55E-11	45	1.207
	inflammatory response	1.55E-09	26	−0.008
	activation of leukocytes	1.96E-07	22	0.392
**Cell Death**	cell death	4.78E-10	75	−0.671
	apoptosis	1.55E-06	54	−1.579
	cell death of immune cells	4.42E-06	21	1.015
	cell death of connective tissue cells	3.69E-05	19	−0.731
	cell death of endothelial cells	5.80E-05	8	−0.904
	neuronal cell death	5.86E-05	19	−2.707
5 **Others**	neoplasia	1.54E-11	82	2.574
	rheumatic disease	1.62E-08	49	−0.104
	arthritis	8.00E-08	46	0.203
	adhesion of connective tissue cells	6.18E-07	10	0.195
	differentiation of connective tissue cells	3.83E-06	18	−0.400
	vasculogenesis	4.90E-06	20	1.897

1 The threshold with FC>0.5 and p<0.05 was used to determine differentially expressed molecules.

2 Category of related biofunctions.

3 The probability that the association between a set of genes in the dataset and a related function is due to random association.

4 The z-score predicts the direction of change for the function. A positive z-score indicates increased function and negative z-score indicates reduced function. An absolute z-score of ≥2 is considered significant.

5 Others includes categories: Cancer, Tissue Development, Cellular Development, Cardiovascular System Development and Function, Skeletal and Muscular Disorders.

**Table 3:** Summary of statistically significant biofunctions associated with the molecules that are differently regulated in *Mmp13^−/−^* granulation tissues at day 14 compared to the corresponding WT samples (IPA Functional Analysis).^1^

*Category* 2	*Function Annotation*	*p-value* 3	*Number of Molecules*	*Regulation z-score* 4
**Cellular Movement**	migration of cells	3.13E-21	86	−1.761
	cell movement	2.95E-19	88	−1.614
	cell movement of leukocytes	3.91E-10	38	−2.275
	cell movement of endothelial cells	7.06E-08	18	0.117
	cell movement of granulocytes	7.16E-08	22	−2.949
	cell movement of smooth muscle cells	6.70E-06	11	−0.304
**Inflammatory Response**	immune response	2.55E-14	66	−0.960
	inflammatory response	3.87E-11	36	−2.078
	chemotaxis of leukocytes	5.36E-07	19	−1.939
	cell movement of neutrophils	9.11E-06	16	−2.300
	quantity of phagocytes	2.20E-05	15	−1.631
**Cell Death**	cell death	3.79E-13	113	0.080
	apoptosis	1.77E-11	90	−0.075
	cell death of blood cells	7.25E-06	28	1.102
	cell death of muscle cells	5.01E-05	15	−0.516
	cell death of connective tissue cells	1.72E-03	21	1.101
**Cellular Growth and Proliferation**	proliferation of cells	1.48E-11	92	−0.230
	growth of cells	1.06E-10	72	−0.705
	proliferation of connective tissue cells	8.19E-08	24	1.485
	proliferation of epithelial cells	7.14E-07	20	0.326
	proliferation of muscle cells	2.09E-05	15	−0.594
**Cardiovascular System Development and Function**	development of blood vessel	1.07E-09	36	−0.663
	angiogenesis	3.63E-09	31	−0.565
	endothelial cell development	2.06E-05	14	−1.283
	proliferation of endothelial cells	1.20E-03	10	−1.827
**Others** 5	tumorigenesis	1.53E-18	138	−0.128
	tissue development	4.63E-11	89	−0.785
	fibrosis	2.21E-10	29	1.226
	vascular disease	4.02E-10	60	−1.196
	differentiation of cells	3.03E-09	66	0.705
	rheumatic disease	6.26E-09	68	−1.800
	arthritis	3.22E-08	64	−1.732
	differentiation of connective tissue cells	5.00E-07	25	0.136
	proteolysis	2.40E-05	14	−2.125
	metabolism of protein	6.75E-05	27	−2.402
	adhesion of immune cells	5.27E-04	15	−2.557

1 The threshold with FC>0.5 and p<0.05 was used to determine differentially expressed molecules.

2 Category of related biofunctions.

3 The probability that the association between a set of genes in the dataset and a related function is due to random association.

4 The z-score predicts the direction of change for the function. A positive z-score ≥2 indicates increased function and negative z-score indicates reduced function. An absolute z-score of ≥2 is considered significant.

5 Others includes categories: Cancer, Tissue Development, Cellular Development, Connective Tissue Disorders, Cardiovascular Disease, Organismal Injury and Abnormalities and Protein Synthesis.

**Table 4:** Summary of statistically significant biofunctions associated with the molecules that are differently regulated in *Mmp13^−/−^* granulation tissues at day 21 compared to the corresponding WT samples (IPA Functional Analysis).^1^

*Category* 2	*Function Annotation*	*p-value* 3	*Number of Molecules*	*Regulation z-score* 4
**Cellular Movement**	migration of endothelial cells	1.84E-10	19	−1.063
	cell movement of tumor cell lines	2.90E-10	32	2.917
	cell movement of muscle cells	1.63E-09	15	1.541
	migration of fibroblast cell lines	4.07E-09	12	1.583
	migration of vascular smooth muscle cells	3.47E-05	7	2.088
**Cell Death**	apoptosis	1.01E-14	87	−3.191
	apoptosis of tumor cell lines	4.55E-10	44	−2.779
	cell survival	4.86E-08	42	2.21
	neuronal cell death	1.37E-05	24	−1.351
	cell death of connective tissue cells	2.35E-05	23	−2.141
	apoptosis of muscle cells	6.28E-04	10	−1.709
**Cellular Growth and Proliferation**	proliferation of cells	6.58E-15	89	2.986
	proliferation of connective tissue cells	3.06E-09	24	2.694
	proliferation of tumor cell lines	3.12E-07	33	2.611
	proliferation of endothelial cells	4.25E-07	14	−0.488
	proliferation of epithelial cells	4.41E-06	17	3.133
**Inflammatory Response**	immune response	6.01E-12	55	0.896
	inflammatory response	8.92E-09	29	1.506
	cell movement of phagocytes	2.74E-08	25	0.839
	chemotaxis of phagocytes	5.06E-06	14	2.594
	chemotaxis of monocytes	9.84E-05	7	2.886
**Cellular Development**	differentiation	8.72E-12	67	1.133
	endothelial cell development	3.27E-10	19	−0.225
	differentiation of connective tissue cells	1.88E-08	25	0.905
**Others** 5	tumorigenesis	1.27E-24	134	−0.432
	angiogenesis	5.37E-11	31	−0.753
	development of organ	1.21E-10	60	0.929
	arthritis	3.86E-09	59	−0.643
	vascularization	1.06E-08	15	1.323
	cellular homeostasis	3.65E-07	41	3.366
	lymphangiogenesis	2.54E-06	6	n.c.

1 The threshold with FC>0.5 and p<0.05 was used to determine differentially expressed molecules.

2 Category of related biofunctions.

3 The probability that the association between a set of genes in the dataset and a related function is due to random association.

4 The z-score predicts the direction of change for the function. A positive z-score indicates increased function and negative z-score indicates reduced function. An absolute z-score of ≥2 is considered significant. n.c., not calculated.

5 Others includes categories: Cancer, Cellular Function and Maintenance, Tissue Development, Connective Tissue Disorders, Organismal Development, Cardiovascular System Development and Function.

**Figure 6:**
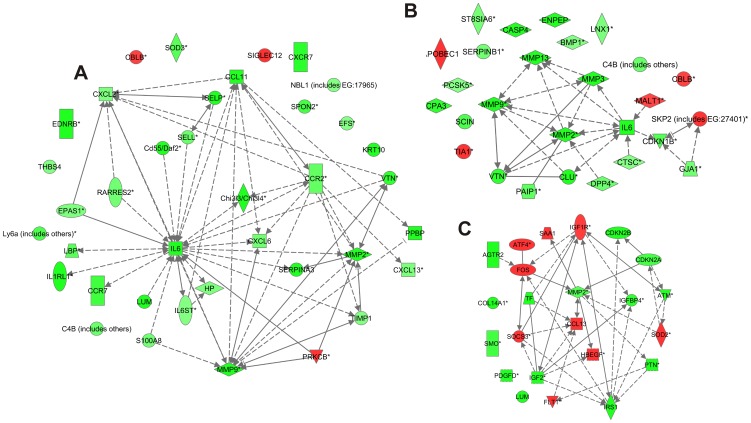
Molecular interactions involved in biological functions cell movement of leukocytes and metabolism of protein at 14 d time point, and proliferation of connective tissue cells at 21 d. The diagrams show the differentially regulated genes involved in biological functions and the molecular interactions based on the literature in Ingenuity Knowledge Base. The expression ratios in the MMP-13 knockout (*Mmp13^−/−^*) granulation tissues compared to WT are visualized as heatmaps. Red color indicates upregulation and green color indicates downregulation in *Mmp13^−/−^* mouse granulation tissues. The intensity of the color implies the magnitude of the FC. The arrows and lines indicate direct (solid line) and indirect (dashed line) functional and physical interactions. The arrows show the direction of regulation. (A) Functional analysis of differentially expressed genes (FC>0.5, *P*<0.05) in *Mmp13^−/−^* granulation tissue compared to WT (14 d) revealed enrichment in the biological function *cell movement of leukocytes* (*P*<3.91E-10), which was predicted to be downregulated in *Mmp13^−/−^* mice (regulation z-score -2.28). (B) Functional analysis was performed as in (A). Enrichment of differentially expressed genes was found in the biological function *metabolism of protein* (*P*<6.75E-05) in *Mmp13^−/−^* granulation tissues, and the function was predicted to be downregulated (regulation z-score -2.40). (C) Functional analysis was performed as in (A). Enrichment of differentially expressed genes was found in the biological function *proliferation of connective tissue cells* at 21 d and the function was predicted to be upregulated in *Mmp13^−/−^* granulation tissue compared to WT (regulation z-score 2.69).

At 14 d, the differentially expressed genes were highly significantly associated with the biofunctions such as *migration of cells*, *immune response*, *cell death*, *proliferation of cells* and *fibrosis* in *Mmp13* ^−/−^ granulation tissue compared to WT (Table 3). Several biofunctions associated with inflammation were significantly down-regulated in *Mmp13* ^−/−^ granulation tissues as compared to WT: *cell movement of leukocytes*, *cell movement of granulocytes*, *cell movement of neutrophils*, *inflammatory response*, and *adhesion of immune cells* (Table 3). The network of molecules involved in the biofunction *cell movement of leukocytes* in the data set (14 d) created with IPA included IL-6, MMP-9, and MMP-2 among other inflammation regulatory molecules (Figure 6A). In accordance with the marked downregulation of several MMPs in *Mmp13* ^−/−^ tissue, functional analysis predicted the biofunctions *proteolysis* and *metabolism of protein* to be significantly downregulated (regulation z-scores -2.13 and -2.40, respectively) (Table 3). The network of molecules involved in the biofunction *metabolism of protein* in the data set (14 d) also included IL-6, as well as MMP-9, and MMP-2, and MMP-3 (Figure 6B). In general, the functional analysis of differentially regulated genes at 14 d time point suggests that inflammation is clearly downregulated in *Mmp13^−/−^* granulation tissue although tissue ingrowth is not significantly delayed at this time point.

Functional analysis of the differentially expressed genes (FC>0.5, *P*-value<0.05) in *Mmp13* ^−/−^ and WT granulation tissue at 21 d, again associated most significantly with the biological functional categories *cellular movement*, *cell death*, *cellular growth and proliferation*, *inflammatory response* and *cellular development* (Table 4). Particularly *apoptosis* and *cell death of connective tissue cells*, were predicted to be significantly downregulated in *Mmp13* ^−/−^ tissue compared to WT (regulation z-score -3.19 and -2.14). In accordance, functions *cell survival* and *proliferation* of different cell types (except endothelial cells), and *cellular homeostasis* were predicted to be significantly upregulated (regulation z-score 2.17 to 3.37). The molecular network of the genes associated with biofunction *proliferation of connective tissue cells* is presented in Figure 6C. Interestingly, at 21 d biofunctions associated with inflammation, *i.e. chemotaxis of phagocytes* and *chemotaxis of monocytes* were predicted to be upregulated (*P*<5.06E-06, regulation z-score 2.59) in *Mmp13^−/−^* tissue. In summary, the functional analysis of differentially regulated genes at 21 d suggests that fibroblast viability and proliferation, as well as inflammation are still active in *Mmp13* ^−/−^ granulation tissue characterized by significantly delayed tissue ingrowth at this time point, whereas in WT, cell apoptosis involved in tissue maturation is increased.

### Comparison of temporal gene expression profiles in WT granulation tissue

To further elucidate the biological processes that are differentially regulated during VCS-induced granulation tissue growth in WT mice, IPA functional analysis was performed on genes showing significant differential expression (FC>1, *P*<0.05) between two consecutive time points. Comparison of granulation tissue at time points 14 d and 7 d revealed altogether 3794 significantly differently expressed genes. Of these, 252 genes displayed FC>1 (upregulated or downregulated expression at 14 vs. 7 d). Correspondingly, comparison of time point 21 d to 14 d in WT mice revealed 2745 differently expressed genes including 252 genes with FC>1.

Functional analysis of gene expression at 14 d and 7 d identified differentially expressed genes associated with the biofunction categories *cellular movement*, *cellular growth and proliferation* and *cell death* in highly significant manner (Table S2). Most notably, biofunctions related to inflammation (*inflammatory response*, and *migration of phagocytes*) showed significant down-regulation based on regulation z-score. The molecular network of differentially expressed genes with highest score (49) included genes for basement membrane molecules *Col4a1* and *Col4a2*, angiogenesis associated *Col8a1* and *Col8a2, Mmp9, Tgfb, Pdgfrb*, myofibroblast associated *Actg2* (γ-SMA) and *Tagln* (Transgelin, Sm22), as well as less abundant fibrillar collagen *Col11a1*, FACIT-collagen *Col12a1* and transmembranous collagen *Col23a1*, which is typically expressed in the epidermis and binds α2β1 integrin [50] (Figure S1A). Based on microarray study, these molecules are upregulated during granulation tissue growth at 14 d compared to 7d. The molecular network with the second highest score (42) included upregulated genes *Mmp13, Mmp3* and also *Mmp11*, *Igfbp*2, *-3* and *-4, Eln* (elastin), *Fbn2* (fibrillin 2), *Fbln1* (fibulin 1) and *Nid2* (nidogen 2). The upregulation of macrophage MARCO receptor and hemoglobin α-chains may reflect macrophage influx into granulation tissue between 7 d and 14 d [43].

Comparison of the gene expression at 21 d to the gene expression at 14 d, the functional analysis identified the biofunctions involved in categories *inflammatory response*, *cellular growth and proliferation*, *cellular movement* and *cell death* as most significantly associated with the differentially expressed genes (Table S3). The biofunctions *inflammation*, *cell movement of monocytes* and *chemotaxis of neutrophils* appeared significantly downregulated at 21 d compared to 14 d. Also, while *contraction of muscle cells* was predicted to be upregulated at 21 d, the *differentiation of muscle cells* appeared significantly downregulated. The most significant molecular networks of the genes differentially expressed at 21 d as compared to 14 d are presented in Figure S2.

### Downregulation of *Mmp2*, *Mmp9*, and *Mmp3* expression in granulation tissue in *Mmp13^−/−^* mice

**Figure 7:**
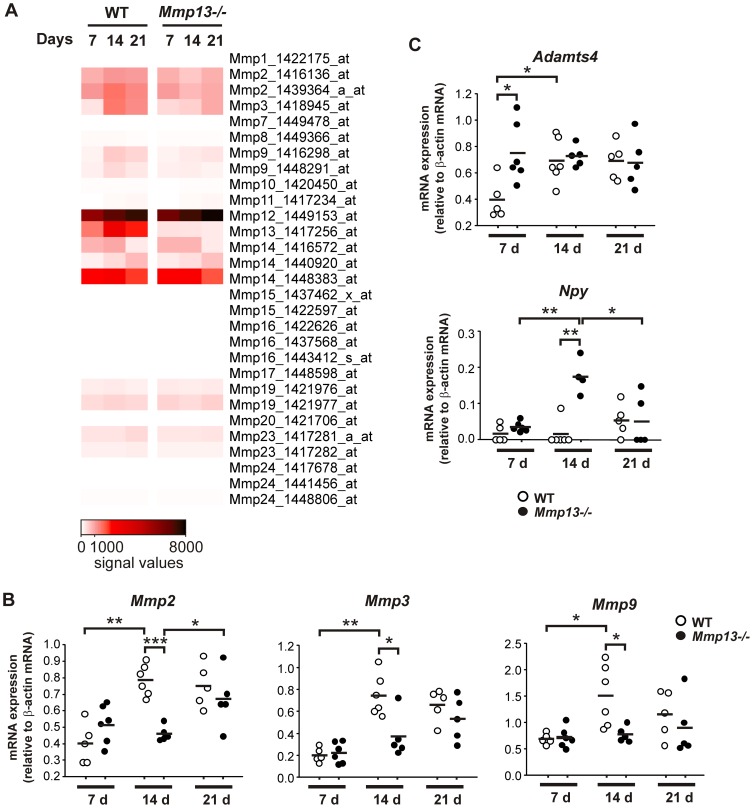
The expression of *Mmp2, Mmp3, Mmp9, Adamts4, and Npy* mRNA in *Mmp13^−/−^* and WT mouse granulation tissue. (**A**) Microarray data of MMP-13 knockout (*Mmp13^−/−^*) and wild type (WT) mouse granulation tissue at 7, 14 and 21 d were analyzed for MMP gene expression, and the signal intensities are illustrated as a heatmap. (**B,C**) Total RNA harvested from WT and *Mmp13^−/−^* granulation tissues at the indicated time points was analyzed for *Mmp2, Mmp3, Mmp9, Adamts4, and Npy* mRNA levels by real-time qRT-PCR. A dot represents a mean of triplicate analysis of a sample with SD≤2% of the mean and the black horizontal bar represents the mean of the experimental replicates. The amplification result of a given mRNA was normalized for β-actin mRNA level in each sample. (*P<0.05, **P<0.001, ***P<0.0001, independent samples T-test, n = 4–6).

A specific temporal expression pattern for several MMPs was observed in WT and *Mmp13* ^−/−^ granulation tissue (Figure 7A). Interestingly, the expression of *Mmp13* in WT mouse granulation tissues was abundant already at 7 d and further upregulation was noted at 14 d and 21 d (Figure 7A). The most notable differences were observed in the expression of *Mmp2*, *Mmp3*, and *Mmp9* mRNAs, which were reduced in *Mmp13* ^−/−^ granulation tissues at 14 d and 21 d time points (Figure 7A). Due to association of these MMPs with angiogenesis, wound contraction and inflammation [3], [4], [51], their expression was further analyzed with real time qRT-PCR. In accordance with the microarray data, the expression of *Mmp2*, *Mmp3*, and *Mmp9* mRNAs were significantly increased in WT mice during the second week of granulation tissue growth (Figure 7B). In contrast, the expression of *Mmp2*, *Mmp3*, and *Mmp9* mRNAs was not markedly altered in *Mmp13* ^−/−^ samples at 14 d compared to 7 d and the expression levels were significantly lower in *Mmp13* ^−/−^ than in WT mouse tissue at 14 d. While the expression of *Mmp2*, *Mmp3*, and *Mmp9* remained at the same level at 21 d time point in WT mice, the expression of *Mmp2* was significantly increased in *Mmp13^−/−^* tissues approaching the levels in WT (Figure 7B). The expression of *Mmp3* and *Mmp9* was not significantly altered in *Mmp13* ^−/−^ mice at 21 d and remained significantly lower than in WT mice (Figure 7B).

### Reduced collagen gel contraction and MMP-2 production by *Mmp13^−/−^* mouse skin fibroblasts

**Figure 8:**
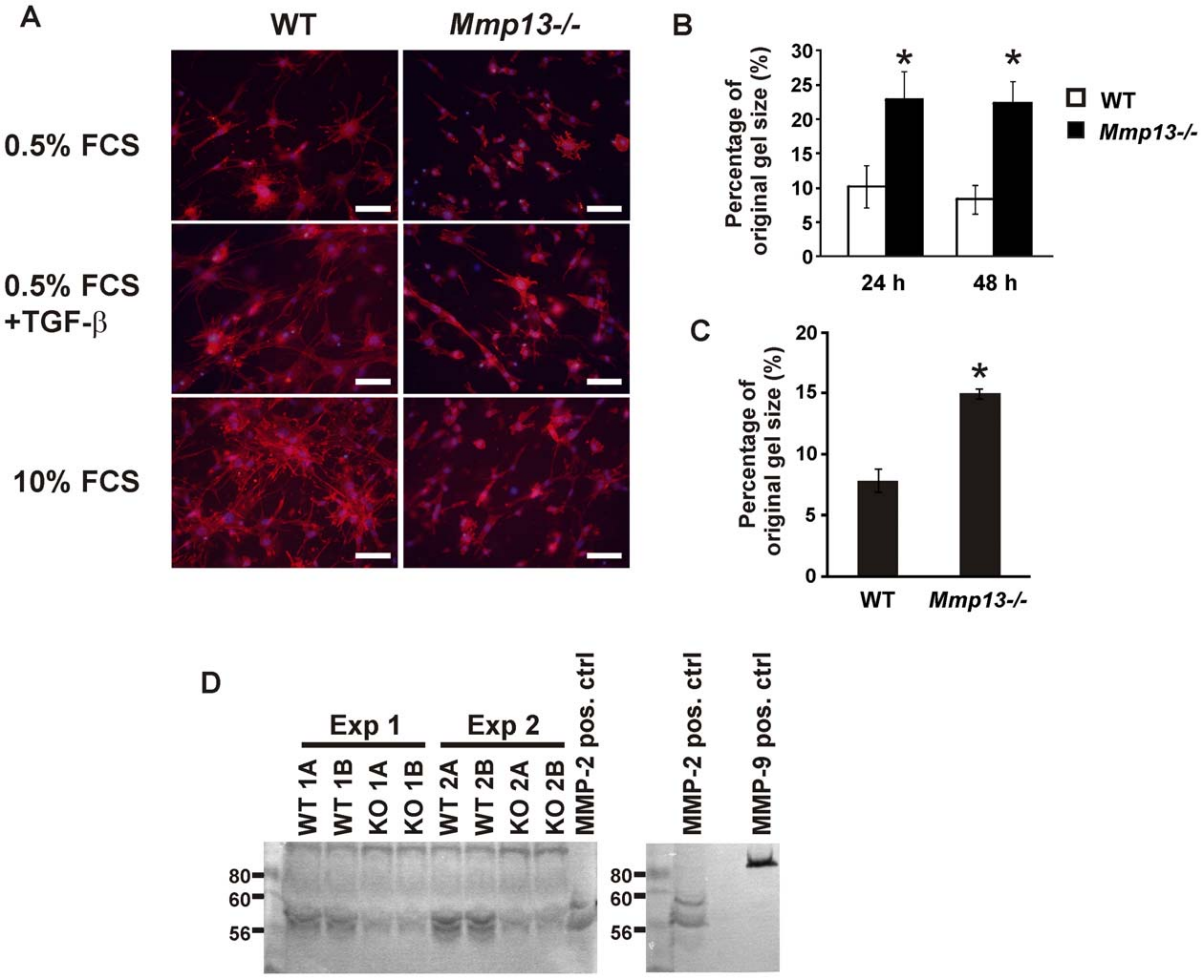
Reduced collagen gel contraction by *Mmp13^−/−^* mouse skin fibroblasts. (**A**) Skin fibroblasts (MSF) established from wild type (WT) and MMP-13 knockout (*Mmp13^−/−^*) mice were cultured in mechanically unloaded (floating) 3D collagen gel at density 2×10^5^/ml for 24 h in the presence of 0.5% FCS, 10% FCS or 0.5% FCS+TGF-β (5 ng/ml), as indicated. The cells were fixed, stained with fluorescently labeled phalloidin and Hoechst, and photographed with 20× magnification to observe morphological appearance. In contrast to *Mmp13^−/−^* MSF, WT fibroblasts displayed stellate morphology with numerous thick cell extensions in response to TGF-β or 10% FCS (Scale bar = 10 µm). (**B**) WT and *Mmp13^−/−^* MSF were cultured in mechanically unloaded 3D collagen gel at density 5×10^5^/ml for 24 and 48 h in the presence of 10% FCS. Contraction of collagen gels was measured from digital images of the gels and is shown as relative to the original gel size. (*P<0.005 compared to control, Independent samples T-test, n = 4) (**C**) WT and *Mmp13^−/−^* MSF were cultured in attached 3D collagen gel at density 5×10^5^/ml for 72 h in the presence of 10% FCS. Subsequently the gels were detached from the well walls and contraction was quantified after 24 h. (*P<0.005 compared to control. Independent samples T-test, n = 3). (**D**) MSF were cultured for 72 h in 3D collagen gel in the presence 10% FCS. Equal aliquots of conditioned media were analyzed in gelatinase zymography.

Contraction of mechanically unloaded 3D collagen gel by fibroblasts reflects their motile activity related to cell adhesion [36]. To address the motile activity of *Mmp13^−/−^* MSF, fibroblasts were seeded in 3D collagenous matrix, and their morphological appearance and collagen contraction capacity were examined. Comparison of WT and *Mmp13^−/−^* MSF cultured in 3D collagen revealed marked differences in cellular morphology. After culturing the fibroblasts for 24 h in relatively low cell density and in low serum, WT MSF formed numerous dendritic cell extensions, whereas in *Mmp13* ^−/−^ MSF the cell extensions were fewer (Figure 8A). Incubation of WT fibroblasts with TGF-β or 10% FCS resulted in stellate morphology characterized by numerous thick cell extensions extending to surrounding ECM and to adjacent cells. In contrast, few cell extensions were noted in *Mmp13* ^−/−^ fibroblasts cultured in the presence of TGF-β or 10% FCS (Figure 8A). In accordance with the altered morphology suggesting reduced cellular contacts, the contraction of collagen gel by *Mmp13^−/−^* MSF was reduced by 60% (*P*<0.005), as compared to WT MSF (Figure 8B).

It has been postulated that restrained collagen gels represent a better model for wound granulation tissue than floating gels, and that the contraction that follows tension dissipation in collagen gel, reflects the mechanical force generated by contraction of fibroblasts [36]. In this respect, WT and *Mmp13* ^−/−^ MSF were allowed to generate mechanical tension into the restrained 3D collagen matrix, and the collagen contraction after stress-relaxation was examined. In accordance with the previous result, WT fibroblasts contracted collagen gel about twice as efficiently as *Mmp13^−/−^* MSF (Figure 8C). These results indicate reduction in motile and contractile activity of *Mmp13^−/−^* MSF, which appears to be due to altered response to serum factors and possibly to TGF-β. When MSF were cultured in a floating 3D collagen gel and in the presence of 10% FCS, WT MSF showed increased MMP-2 production compared to *Mmp13^−/−^* MSF (Figure 8D).

### Upregulation of *Adamts4* and *Npy* expression in granulation tissue of *Mmp13^−/−^* mice

Microarray analysis revealed marked upregulation of *Adamts4* and *Npy* in *Mmp13^−/−^* granulation tissue at 7 d and 14 d time points, respectively (Figure 4A). As these two genes have been linked to angiogenesis [40]–[42], [51], their expression in granulation tissue was verified by qRT-PCR. The level of *Adamts4* mRNA was significantly upregulated in *Mmp13^−/−^* granulation tissue compared to WT mice at 7 d (Figure 7C). However, at 14 d and 21 d the expression of *Adamts4* mRNA increased in WT tissue and no marked difference between WT and *Mmp13^−/−^* mice was detected (Figure 7C).

The expression level of *Npy* mRNA was low at 7 d in both WT and *Mmp13^−/−^* tissue (Figure 7C). However, a significant upregulation was observed at 14 d in *Mmp13^−/−^* tissue, as compared to 7 d and to WT tissue (Figure 7C). At 21 d, the expression of *Npy* was again downregulated in *Mmp13^−/−^* tissue and was similar as in WT mice. As ADAMTS-4 and NPY are both implicated in blood vessel formation [40]–[42], [51], the results are interesting with respect to the increased density of microvessels observed by IHC at 14 d in *Mmp13* ^−/−^ granulation tissue.

## Discussion

MMPs are important players at all stages in cutaneous wound repair [3], [4]. They play a role in the clearance of ECM barriers, in the release and activation of various bioactive proteins as well as in the regulation of cell-ECM interactions [3], [4]. In humans, MMP-13 is not detected in normally healing adult skin wounds, although it is expressed by fibroblasts in human fetal skin wounds characterized by rapid healing with minimal scar [23], [25]. In mouse skin wounds, which also typically tend to heal rapidly and do not develop scars comparable to humans, MMP-13 is suggested to function analogously to MMP-1 in adult human skin wound [9], [12], [21], [22]. However, the mechanistic role of mouse MMP-13 in the remodeling of wound ECM remains unclear.

In this study, we have examined the specific role of MMP-13 in granulation tissue formation by utilizing a well established experimental model of granulation tissue induction by viscose cellulose sponge (VCS) [29], [31]. This model allows precise examination of various parameters involved specifically in granulation tissue formation, such as tissue growth and angiogenesis. Using this model in MMP-13 deficient (*Mmp13^−/−^*) mouse strain, we noted that MMP-13 is essential for normal generation of granulation tissue in mice. As assessed by histological parameters, we first observed a pronounced reduction in the growth of cellular granulation tissue into VCS in *Mmp13^−/−^* mice during the third postoperative week at day 21, when *Mmp13^−/−^* mice showed less than 60% of the cellular tissue ingrowth, as compared to WT mice.

A marked alteration in the orientation of myofibroblasts in the early *Mmp13* ^−/−^ granulation tissue was also noted at day 7. Interestingly, MMP-13 has recently been shown to play a role in myofibroblast differentiation *in vitro*, suggested to be due to the activation of TGF-β [28]. In the same study, reduction in myofibroblast number and wound contraction was detected in *Mmp13^−/−^* mice at the termination of epithelialization [28]. Our results show that MMP-13 augments myofibroblast function as defined by initial parallel assembly of cell masses important for the contraction. Moreover, with respect to myofibroblast activation, lack of MMP-13 activity appears to be compensated *in vivo*, since efficient alignment of myofibroblasts comparable to WT mice was noted in *Mmp13^−/−^* granulation tissue by day 14. Interestingly, at 21 d, when prominent difference in granulation tissue growth was also noted, α-SMA positive cells were still abundant in *Mmp13^−/−^* tissue, while their number was already diminishing in corresponding areas in WT mouse granulation tissue. This observation was supported by gene expression profiling showing decreased biofunctions in *apoptosis*, including *apoptosis of connective tissue cells* and *apoptosis of muscle cells*, in *Mmp13^−/−^* mouse granulation tissue at 21 d. However, myofibroblasts were still abundantly present in *Mmp13* ^−/−^ tissue in the areas with strong staining of fibrous ECM characterizing more matured ECM. Thus, the observation rather suggests that myofibroblasts are unable to move towards inner parts of VCS implant during delayed granulation tissue growth *Mmp13* ^−/−^ mice.

The experiments with mouse skin fibroblasts cultured within 3D collagen indicated that processing of TGF-β and possibly other serum factors by MMP-13 are needed to induce morphological changes that suggest enhanced cell adhesion and cytoskeletal activity. This is associated with fibroblast-mediated collagen gel contraction, which depending on the model system reflects motile (floating gels) or contractile (restrained-relaxed gel) activity of fibroblasts. In this study, both models showed decreased collagen gel contraction by *Mmp13^−/−^* fibroblasts. Thus, MMP-13 may augment fibroblast penetration into VCS by enhancing their motile activity and it may also increase contractile force generated in fibroblasts. The activity of MMP-13 may also affect cell adhesion to matrix and to adjacent cells, which could also be related to defective assembly of *Mmp13^−/−^* myofibroblasts detected at 7 d *in vivo*.

Stromal expression of MMP-13 has been implicated in angiogenesis of malignant melanoma and cutaneous SCC [52], [53] and lack of MMP-13 was reported to reduce vascular density of wound granulation tissue [28]. In addition, MMP-13 has recently been implicated in corneal vascularization [54]. In the present study, significantly higher density of small vessels in *Mmp13^−/−^* granulation tissue was detected at day 14, apparently indicating enhanced angiogenesis. Accordingly, two interesting genes involved in angiogenesis, *Adamts4* [51] and *Npy* [40]–[42], were upregulated in *Mmp13^−/−^* granulation tissues at 7 and 14 d, respectively, suggesting they could be candidate genes implicated in increased microvessel density. Another pronounced difference between the genotypes in terms of vascularization was the virtual absence of large vessels at day 21 d in *Mmp13^−/−^* mouse tissue. Functional analysis of the global gene expression data suggested some increase in *vasculogenesis* at 7 d, which supports the observations on the increased microvasculature at 14 d in *Mmp13^−/−^* granulation tissue. Despite of upregulation of angiogenic genes *Npy, Fgf13, Met* and *Cyr61* [40], in *Mmp13^−/−^* granulation tissue at 14 d, the functional analysis of the gene expression data suggested downregulation of the process at 14 d and at 21 d. This reflects the difference noted in the amount of large vessels at histological level at 21 d time point. It is possible, that the presence of large blood vessels in WT mouse granulation tissue at 21 d was not a result of increased tissue growth, but that the large vessels were required for proper granulation tissue growth.

Genome wide transcriptional profiling studies of various wound healing models in mice and humans have revealed hundreds of differentially regulated genes in different stages of wound healing. These include genes involved in inflammatory response, pathogen recognition, endopeptidase activity, ECM composition and various regulatory processes in cells [55]–[57]. Although laser microdissection has enabled analysis of gene expression profiles in specific cells [57], the majority of wound microarray studies have not discriminated the genes expressed by epithelial cells from the genes expressed by granulation tissue cells. Here, we performed global gene expression profiling specifically in granulation tissue cells excluding epidermal keratinocytes and adjacent intact tissue. The data were analyzed with respect to the genotype and to the temporal alterations in gene expression.

The global gene expression analysis comparing the 7 d samples revealed several interesting genes, which were upregulated in *Mmp13^−/−^* granulation tissue, such as angiogenic *Adamts4* [51] and growth inhibitory *Ifi202b* and *Cadm1* [37], [38], [46]. However, significant up- or downregulation of biological functions between genotypes at 7 d was noted only with respect to *neuronal cell death* and *neoplasia*. The most obvious differences between the genotypes were observed at 14 d time point, which is in accordance with the fact that the difference in granulation tissue growth was not obvious until during the third week. At day 14, a significant downregulation of different aspects of inflammatory reaction were observed. *Il6*, which was markedly downregulated in *Mmp13^−/−^* granulation tissue at 7 d and 14 d is one of the key molecules regulating biofunctions, such as *cell motility of leucocytes* and *metabolism of protein*. In fact, decreased *proteolysis* detected in *Mmp13^−/−^* tissue may be partially due to the impaired regulation of the composition of the inflammatory cells. Also, as inflammatory cells are a major source of chemotactic molecules during wound healing [2], deprivation of these molecules could severely interfere with fibroblast migration in wound tissue. These results provide evidence that in mouse wound, MMP-13 activity regulates functions pivotal for tissue growth. Moreover, these results suggest for the first time a role for MMP-13 in the positive regulation of the inflammatory processes involved in wound healing. Interestingly, at 21 d, the biofunctions *chemotaxis of phagocytes* and *proliferation of cells* were markedly upregulated in *Mmp13^−/−^* granulation tissue, whereas *apoptosis* was downregulated. These results suggest that although inflammation and growth of granulation tissue is delayed in *Mmp13* ^−/−^ mice, it is restored at later stage.

Comparison of the gene expression profiles of the two genotypes at specific time points during granulation tissue development suggested marked downregulation of several MMPs in *Mmp13^−/−^* tissue. Increased expression of MMP-8 (neutrophil collagenase) has been suggested to cover the lack of the proteolytic activity of MMP-13 in skin wound in MMP-13 deficient mice [27]. However, in the granulation tissue model used in this study, no signs of enzymatic redundancy were detected, as the expression of other collagenolytic MMPs (*Mmp2, Mmp8*, and *Mmp14*) was not induced in *Mmp13* ^−/−^ granulation tissues. Particularly, the expression of *Mmp2*, *Mmp3*, *Mmp9*, and *Mmp13* were upregulated during the second week of granulation tissue growth in WT mice (Figure 7A). *Mmp13* ^−/−^ granulation tissue did not display similar upregulation pattern resulting in significant differences in the expression levels of *Mmp2*, *Mmp3*, and *Mmp9*. Thus, MMP-13 may increase proteolytic potential in the granulation tissue and ultimately regulate a variety of additional biological processes *via* activation of other bioactive molecules or alteration in the matrix composition. As MMP-2 is associated with fibroblast-mediated matrix remodeling [58], which typically involves cellular movement, it could promote population of VCS by fibroblasts. In addition, MMP-13 may regulate angiogenesis *via* stimulating *Mmp2* and *Mmp9* expression [59], [60]. Here, the temporal induction of *Mmp2* and *Mmp9* expression correlated with the appearance of large blood vessels in WT. MMP-13 has recently been proposed to release hepatocyte growth factor (HGF) subsequently resulting in MMP-9 induction providing a potential mechanism for MMP-13-mediated upregulation of MMP-9 [61]. Moreover, upregulation of HGF receptor *Met* expression in *Mmp13^−/−^* granulation tissue at all the time points (Figure 5) may reflect disability of HGF function, supporting this hypothesis.

Among the genes involved in cellular angiogenic, mitogenic and locomotive activity, the expression of *Pdgfd* was significantly downregulated in *Mmp13^−/−^* granulation tissue at all time points. PDGF-D has been shown to stimulate proliferation of fibroblasts *in vitro*, enhance activation of tissue macrophages and stabilize blood vessels [44]. Recently, PDGF-D was reported to positively regulate cancer related angiogenesis, cell growth and invasion, and the expression of MMP-9 and VEGF by pancreatic cancer cells [45]. The observations that *Mmp9* and VEGF-D (*Figf*) were both downregulated in *Mmp13^−/−^* granulation tissue at 14 and 21 d, respectively, and the receptor of PDGF-D, *Pdgfrb*, was upregulated at 7 d, suggest the biological importance of the observation. Thus, reduced expression of PDGF-D may provide a possible mechanism for reduced growth and altered blood vessel pattern observed in *Mmp13^−/−^* granulation tissue in later stage. Also, activation of TGF-β by MMP-13 provides a putative explanation for our observations, since TGF-β has been implicated in the induction of several MMPs including MMP-9 [62] and MMP-13 [25], and in inhibition of the expression of ADAMTS-4 in human macrophages [63]. However, it is of note that we could not identify dysregulation of TGF-β signaling molecules at the examined time points.

In conclusion, the data presented here provide evidence for the important role of MMP-13, a multifunctional proteinase, in regulating multiple cellular functions including myofibroblast activity, cell motility, angiogenesis inflammation, and proteolysis during growth and maturation of wound granulation tissue. In addition, the results of the present study suggest possible mechanisms of physiological function of MMP-13 in human fetal skin wounds, and they can be employed in development of novel therapeutic modalities for promoting impaired wound repair and inhibiting excessive scar formation. Human MMP-13 and MMP-1 are also linked to cutaneous malignancies, such as SCC, and melanoma [64], [65], which involve inflammation, stromal cell activation, and angiogenesis in the tumor micro-environment. It is therefore conceivable, that the results presented in this study also provide mechanistic insight into the role of MMP-13 and MMP-1 in the progression of these malignant tumors.

## Supporting Information

Figure S1 **Molecular networks of differentially expressed genes in WT granulation tissues at 14 d as compared to 7 d.** The molecular networks were created by IPA software. (**A**) The molecular network with the highest significance is composed of the molecules shown (score 49). (**B**) The molecular network with the second highest significance is composed of the molecules shown (score 42). The diagrams show the molecular networks of differentially regulated genes and the molecular interactions based on the literature in Ingenuity Knowledge Base. MMPs are highlighted in yellow. The expression ratios are visualized as heatmap. Red color indicates upregulation and green color indicates downregulation. Only the molecules with FC>1 and *P*-value<0.05 are colored in red or green. Gray color indicates FC<1. The arrows and lines indicate direct (solid line) and indirect (dashed line) functional and physical interactions. The arrows show the direction of regulation.
(TIF)

Figure S2 **Molecular networks of differentially expressed genes in WT granulation tissues at 21 d as compared to 14 d.** The molecular networks were created by IPA software. (**A**) The molecular network with the highest significance is composed of the molecules shown (score 44). (**B**) The molecular network with the second highest significance is composed of the molecules shown (score 34). The diagrams show the molecular networks of differentially regulated genes and the molecular interactions based on the literature in Ingenuity Knowledge Base. The expression ratios are visualized as heatmap. Red color indicates upregulation and green color indicates downregulation. Only the molecules with FC>1 and *P*-value <0.05 are colored in red or green. Gray color indicates FC<1. The arrows and lines indicate direct (solid line) and indirect (dashed line) functional and physical interactions. The arrows show the direction of regulation.
(TIF)

Table S1 **Sequences of primers and probes used for quantitative RT-PCR.**
(DOC)
(TIF)

Table S2 **Summary of statistically significant biofunctions associated with the molecules that are differently regulated at day 14 compared to day 7 in WT samples (IPA Functional Analysis).**
(DOC)

Table S3 **Summary of statistically significant biofunctions associated with the molecules that are differently regulated at day 21 compared to day 14 in WT samples (IPA Functional Analysis).^1^**
(DOC)
