# Supplementary figures and images for: MMP-13 Regulates Growth of Wound Granulation Tissue and Modulates Gene Expression Signatures Involved in Inflammation, Proteolysis, and Cell Viability
Source: PLoS One. 2012 Aug 7;7(8):e42596. doi: 10.1371/journal.pone.0042596 (PMC3413640; doi:10.1371/journal.pone.0042596)

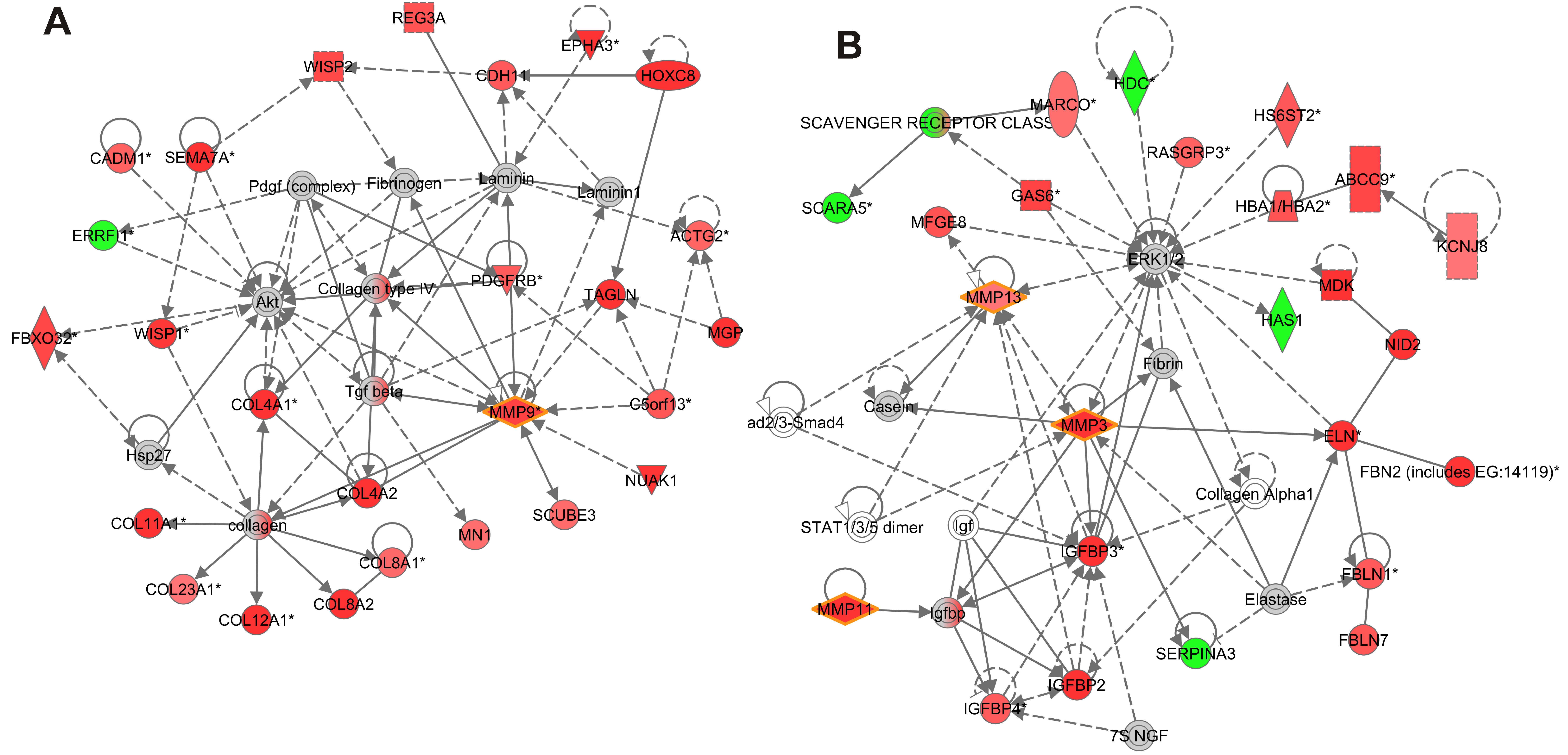

Supplement: Figure S1 — Molecular networks of differentially expressed genes in WT granulation tissues at 14 d as compared to 7 d. The molecular networks were created by IPA software. (A) The molecular network with the highest significance is composed of the molecules shown (score 49). (B) The molecular network with the second highest significance is composed of the molecules shown (score 42). The diagrams show the molecular networks of differentially regulated genes and the molecular interactions based on the literature in Ingenuity Knowledge Base. MMPs are highlighted in yellow. The expression ratios are visualized as heatmap. Red color indicates upregulation and green color indicates downregulation. Only the molecules with FC>1 and P-value<0.05 are colored in red or green. Gray color indicates FC<1. The arrows and lines indicate direct (solid line) and indirect (dashed line) functional and physical interactions. The arrows show the direction of regulation. (TIF) [file pone.0042596.s001.tif]

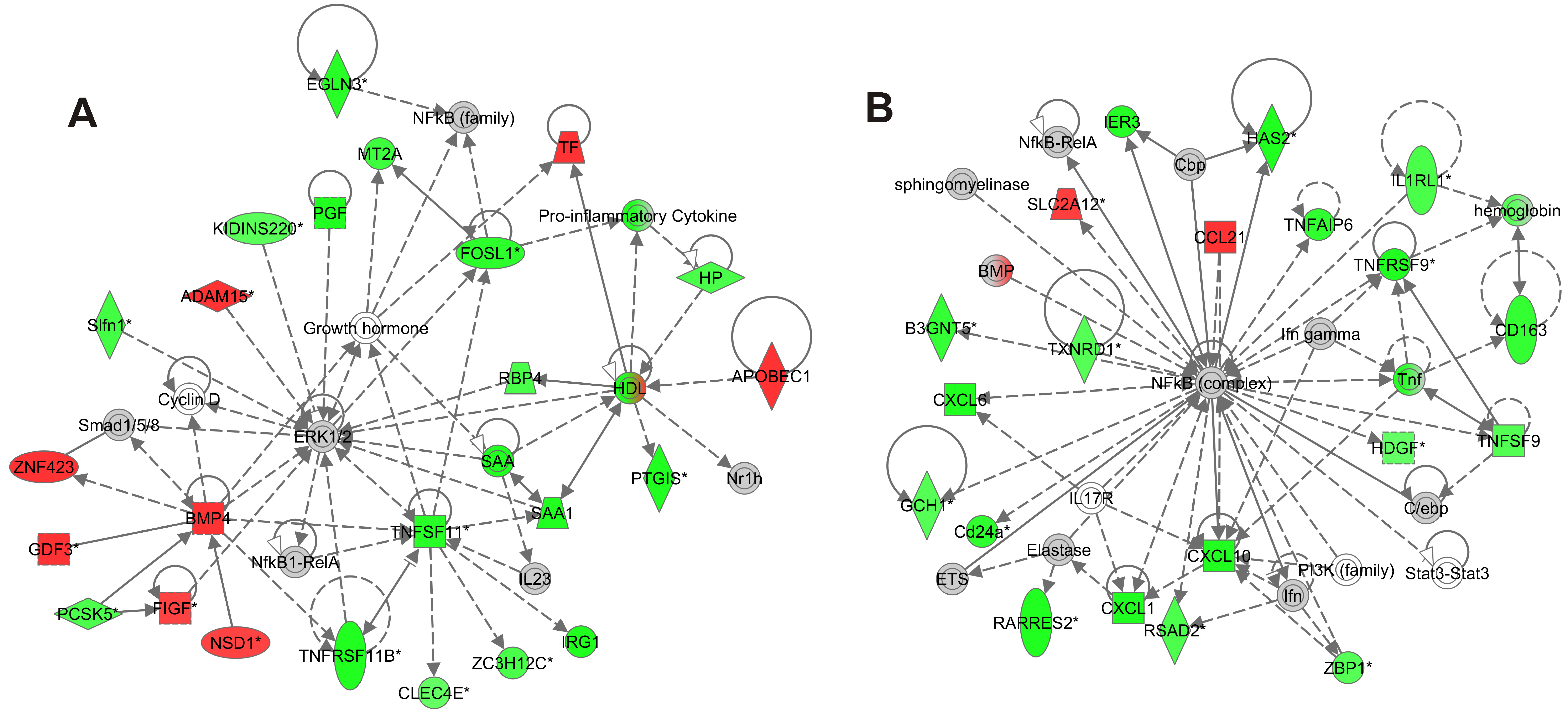

Supplement: Figure S2 — Molecular networks of differentially expressed genes in WT granulation tissues at 21 d as compared to 14 d. The molecular networks were created by IPA software. (A) The molecular network with the highest significance is composed of the molecules shown (score 44). (B) The molecular network with the second highest significance is composed of the molecules shown (score 34). The diagrams show the molecular networks of differentially regulated genes and the molecular interactions based on the literature in Ingenuity Knowledge Base. The expression ratios are visualized as heatmap. Red color indicates upregulation and green color indicates downregulation. Only the molecules with FC>1 and P-value <0.05 are colored in red or green. Gray color indicates FC<1. The arrows and lines indicate direct (solid line) and indirect (dashed line) functional and physical interactions. The arrows show the direction of regulation. (TIF) [file pone.0042596.s002.tif]
