# Supplementary material for: MMP-13 Regulates Growth of Wound Granulation Tissue and Modulates Gene Expression Signatures Involved in Inflammation, Proteolysis, and Cell Viability
Source: PLoS One. 2012 Aug 7;7(8):e42596. doi: 10.1371/journal.pone.0042596 (PMC3413640; doi:10.1371/journal.pone.0042596)
Supplement: Table S1 — Sequences of primers and probes used for quantitative RT-PCR. (DOC) (TIF) [file pone.0042596.s003.doc]

**Table S1.** Sequences of primers and probes used for quantitative RT-PCR.

| **mβ-actin** | forward | 5'-TGGCTCCTAGCACCATGAAGA-3' |
| --- | --- | --- |
|  | reverse | 5'-GTGGACAGTGAGGCCAGGAT-3' |
|  | probe | FAM-5’-CAAGATCATTGCTCCTCCTGAGCGCA-3’-TAMRA |
|  |  |  |
| **mMMP-2** | forward | 5’-ctgcaccatcgcccatcatc-3’ |
|  | reverse | 5’-gcactgccaactctttgtctg-3’ |
|  | probe | FAM-5’-aagttccccggcgatgtcgcc-3’-BHQ |
|  |  |  |
| **mMMP-9** | forward | 5’-CCTGGCTCTCCTGGCTTTC-3’ |
|  | reverse | 5’-GTCCGTGAGGTTGGAGGTTT-3’ |
|  | probe | FAM-5’-CTGCAGCTCTGCTGCCCCTTA-3’-BHQ |
|  |  |  |
| **mMMP-3** | forward | 5’-TGGACAGAGGATGTCACTGGTA-3’ |
|  | reverse | 5’-GCCTTGGCTGAGTGGTAGAG-3’ |
|  | probe | FAM-5’-AACCTATTCCTGGTTGCTGCTCATGAA-3’-BHQ |
|  |  |  |
| **mADAMTS-4** | forward | 5’- ATGGGCTTGCATCCCAGGAG -3’ |
|  | reverse | 5’- GCCACTGCACAGTGTGAA -3’ |
|  | probe | FAM-5’-ACTGGGCACTGGCTGCGAAGAT-3’-BHQ |
