# Supplementary material for: MMP-13 Regulates Growth of Wound Granulation Tissue and Modulates Gene Expression Signatures Involved in Inflammation, Proteolysis, and Cell Viability
Source: PLoS One. 2012 Aug 7;7(8):e42596. doi: 10.1371/journal.pone.0042596 (PMC3413640; doi:10.1371/journal.pone.0042596)
Supplement: Table S2 — Summary of statistically significant biofunctions associated with the molecules that are differently regulated at day 14 compared to day 7 in WT samples (IPA Functional Analysis). (DOC) [file pone.0042596.s004.doc]

**Table S2.** Summary of statistically significant biofunctions associated with the molecules that are differently regulated at day 14 compared to day 7 in WT samples (IPA Functional Analysis) .^1^

| *Category^2^* | *Function Annotation* | *p-value^3^* | *Number of Molecules* | *Regulation z-score^4^* |
| --- | --- | --- | --- | --- |
| Cellular Movement P-value 6.22E-20 - 8.84E-04 | cell movement | 6.22E-20 | 71 | -0.893 |
|  | cell movement of myeloid cells | 3.77E-14 | 30 | -1.554 |
|  | chemotaxis of leukocytes | 8.46E-09 | 18 | -1.178 |
|  | migration of phagocytes | 1.55E-07 | 15 | -2.248 |
| Cardiovascular System Development and Function P-value 4.69E-19 - 8.84E-04 | vasculogenesis | 4.69E-19 | 39 | -1.979 |
|  | angiogenesis | 2.89E-18 | 37 | -1.16 |
|  | cell movement of endothelial cells | 1.90E-10 | 18 | -1.192 |
|  | endothelial cell development | 5.29E-09 | 16 | -0.897 |
|  | vascularization | 5.94E-09 | 14 | -0.74 |
| Cellular Growth and Proliferation P-value 1.23E-14 - 8.09E-04 | proliferation of cells | 1.23E-14 | 77 | 0.271 |
|  | proliferation of smooth muscle cells | 9.19E-08 | 14 | -0.551 |
|  | proliferation of epithelial cells | 2.59E-07 | 17 | -1.598 |
|  | proliferation of connective tissue cells | 9.80E-07 | 18 | -0.2 |
|  | proliferation of endothelial cells | 1.70E-06 | 12 | -0.914 |
| Inflammatory Response P-value 5.90E-14 - 5.5E-05 | cell movement of phagocytes | 5.90E-14 | 30 | -1.072 |
|  | immune response | 3.67E-11 | 47 | -0.437 |
|  | inflammatory response | 7.13E-11 | 29 | -2.072 |
|  | chemotaxis of leukocytes | 8.46E-09 | 18 | -1.178 |
|  | cell movement of monocytes | 9.15E-09 | 13 | -1.022 |
|  | cell movement of neutrophils | 6.34E-08 | 16 | -1.201 |
|  | migration of phagocytes | 1.55E-07 | 15 | -2.248 |
| Cell Death P-value 5.03E-12 - 8.00E-04 | apoptosis | 8.21E-13 | 72 | -0.82 |
|  | apoptosis of epithelial cells | 1.12E-05 | 11 | 2.258 |
|  | cell death of muscle cells | 1.46E-05 | 13 | 0.428 |
|  | cell survival | 2.01E-05 | 31 | -0.516 |
| Others^5^ | neoplasia | 5.26E-18 | 100 | 1.595 |
|  | development of connective tissue | 2.78E-12 | 34 | -0.716 |
|  | organogenesis | 1.20E-10 | 53 | 0.357 |
|  | quantity of cells | 3.97E-09 | 40 | 1.372 |
|  | proliferation of vascular smooth muscle cells | 7.11E-05 | 7 | 0.327 |
|  | contraction of smooth muscle | 1.02E-03 | 5 | -0.603 |
| ^1^The threshold with FC>1 and p<0.05 was used to determine differentially expressed molecules. | | | | |
| ^2^Category of related biofunctions. | | | | |
| ^3^The probability that the association between a set of genes in the dataset and a related function is due to random association. | | | | |
| ^4^ The z-score predicts the direction of change for the function. A positive z-score indicates increased function and negative z-score indicates reduced function. An absolute z-score of ≥ 2 is considered statistically significant. | | | | |
| ^5^Others includes categories: Cancer, Tissue Development, Embryonic Development, Tissue Morphology and Skeletal and Muscular System Development and Function. | | | | |
