# Supplementary material for: MMP-13 Regulates Growth of Wound Granulation Tissue and Modulates Gene Expression Signatures Involved in Inflammation, Proteolysis, and Cell Viability
Source: PLoS One. 2012 Aug 7;7(8):e42596. doi: 10.1371/journal.pone.0042596 (PMC3413640; doi:10.1371/journal.pone.0042596)
Supplement: Table S3 — Summary of statistically significant biofunctions associated with the molecules that are differently regulated at day 21 compared to day 14 in WT samples (IPA Functional Analysis).1 (DOC) [file pone.0042596.s005.doc]

**Table S3.** Summary of statistically significant biofunctions associated with the molecules that are differently regulated at day 21 compared to day 14 in WT samples (IPA Functional Analysis) .^1^

| *Category^2^* | *Function Annotation* | *p-value^3^* | *Number of Molecules* | *Regulation z-score^4^* |
| --- | --- | --- | --- | --- |
| Inflammatory Response P-value 8.79E-17 - 6.84E-04 | immune response | 8.79E-17 | 57 | -0.868 |
|  | inflammatory response | 4.09E-15 | 35 | -0.834 |
|  | cell movement of phagocytes | 5.68E-13 | 29 | -0.921 |
|  | chemotaxis of leukocytes | 6.12E-13 | 23 | -1.094 |
|  | chemotaxis of phagocytes | 2.80E-11 | 19 | -1.528 |
|  | activation of phagocytes | 1.28E-09 | 18 | -1.92 |
|  | inflammation | 1.26E-08 | 22 | -2.303 |
|  | transmigration of neutrophils | 6.74E-08 | 7 | 1.654 |
|  | cell movement of monocytes | 6.21E-06 | 10 | -2.259 |
|  | chemotaxis of neutrophils | 7.97E-05 | 8 | -2.051 |
| Cellular Growth and Proliferation P-value 8.02E-17 - 6.84E-04 | proliferation of cells | 8.02E-17 | 82 | -0.02 |
|  | growth of cells | 6.02E-11 | 57 | -1.446 |
|  | proliferation of endothelial cells | 4.14E-08 | 14 | -0.381 |
|  | proliferation of connective tissue cells | 5.72E-08 | 20 | -0.188 |
|  | proliferation of muscle cells | 3.81E-05 | 12 | -1.771 |
|  | expansion of blood cells | 3.87E-05 | 10 | -2.094 |
| Cellular Movement P-value 1.21E-17 - 6.97E-04 | migration of cells | 1.26E-17 | 64 | -0.28 |
|  | leukocyte migration | 7.89E-14 | 39 | -0.931 |
|  | cell movement of endothelial cells | 5.74E-07 | 14 | 0.302 |
|  | migration of fibroblast cell lines | 9.14E-07 | 9 | -0.557 |
| Cell Death P-value 1.95E-14 - 6.97E-04 | apoptosis | 1.95E-12 | 72 | 1.313 |
|  | cell viability | 4.36E-06 | 15 | -1.339 |
|  | apoptosis of muscle cells | 4.86E-06 | 12 | 0.462 |
|  | cell death of immune cells | 5.18E-06 | 22 | 0.18 |
|  | cell death of connective tissue cells | 1.22E-05 | 21 | 1.478 |
| Others^5^ | tumorigenesis | 1.35E-19 | 109 | 0.107 |
|  | fibrosis | 2.77E-14 | 29 | -1.516 |
|  | muscle contraction | 8.97E-12 | 19 | n.c. |
|  | differentiation of connective tissue cells | 3.82E-10 | 25 | -1.756 |
|  | quantity of cells | 5.86E-10 | 42 | 0.747 |
|  | endothelial cell development | 6.67E-09 | 16 | 0.559 |
|  | angiogenesis | 6.76E-09 | 25 | -0.016 |
|  | contraction of muscle cells | 7.64E-06 | 6 | 2.083 |
|  | differentiation of muscle cell lines | 4.48E-05 | 8 | -2.457 |
| ^1^The threshold with FC>1 and p<0.05 was used to determine differentially expressed molecules. | | | | |
| ^2^Category of related biofunctions. | | | | |
| ^3^The probability that the association between a set of genes in the dataset and a related function is due to random association. | | | | |
| ^4^ The z-score predicts the direction of change for the function. A positive z-score indicates increased function and negative z-score indicates reduced function. An absolute z-score of ≥ 2 is considered statistically significant. n.c., not calculated. | | | | |
| ^5^Others includes categories: Cancer, Cardiovascular System Development and Function, Cellular development, Organismal Injury and Abnormalities, Tissue Morphology and Skeletal and Muscular System Development and Function. | | | | |
